# Genome-Wide DNA Methylation Profiles Reveal Common Epigenetic Patterns of Interferon-Related Genes in Multiple Autoimmune Diseases

**DOI:** 10.3389/fgene.2019.00223

**Published:** 2019-04-05

**Authors:** Sidi Chen, Weilin Pu, Shicheng Guo, Li Jin, Dongyi He, Jiucun Wang

**Affiliations:** ^1^State Key Laboratory of Genetic Engineering, Collaborative Innovation Center for Genetics and Development, School of Life Sciences, Fudan University, Shanghai, China; ^2^Human Phenome Institute, Fudan University, Shanghai, China; ^3^Center for Precision Medicine Research, Marshfield Clinic Research Institute, Marshfield, WI, United States; ^4^Department of Rheumatology, Shanghai Guanghua Hospital of Integrated Traditional and Western Medicine, Shanghai, China; ^5^Arthritis Institute of Integrated Traditional and Western Medicine, Shanghai Traditional Chinese Medicine Research Institute, Shanghai, China; ^6^Institute of Rheumatology, Immunology and Allergy, Fudan University, Shanghai, China

**Keywords:** autoimmune diseases, DNA methylation, type I interferon, biomarker, prediction

## Abstract

Graves’ disease (GD), rheumatoid arthritis (RA), systemic lupus erythematosus (SLE) and systemic sclerosis (SSc) are complex autoimmune diseases sharing common clinical, genetic and pathogenetic features. However, the commonalities of the DNA methylation profiles for these diseases are still unknown. We conducted an integrative analysis of the multiple-autoimmune disease methylation dataset including GD, RA, SLE, and SSc samples, to identify the common methylation patterns of autoimmune diseases. We identified 15,289 differentially methylated sites between multiple-autoimmune disease patients and controls in CD4+ T cells. We found that the most significant differentially methylated sites had a remarkable enrichment in type I interferon (IFN) pathway genes. Similarly, we identified 9,295 differentially methylated sites between GD/SSc patients and controls in CD8+ T cells. The overall IFN-related gene panel annotated by gene ontology (GO) showed an excellent diagnostic capacity in CD4+ T cells (Sensitivity = 0.82, specificity = 0.82 and AUC = 0.90), while *IFI44L*, another IFN-related gene not annotated by GO, showed high prediction ability in both CD4+ (AUC = 0.86) and CD8+ (AUC = 0.75) T cells. In conclusion, our study demonstrated that hypomethylation of IFN-related genes is a common feature of GD/RA/SLE/SSc patients in CD4+ T cells, and the DNA methylation profile of IFN-related genes could be promising biomarkers for the diagnosis of GD, RA, SLE, and SSc.

## Introduction

Autoimmune diseases are multifactorial complex diseases characterized by loss of immunologic tolerance to self-antigens, inappropriate activation of autoimmune responses and damage of target organ systems (Wandstrat and Wakeland, 2001). Currently, over 100 types of autoimmune diseases affect 5–10% of the population worldwide and are one of the key causes of morbidity and mortality. Moreover, the economic burden of autoimmune diseases is enormous due to high rates of disability and comorbidity as well as increasing treatment costs (Shoenfeld et al., 2008). However, the understanding of autoimmune diseases is still limited; the exact etiology and pathogenesis remains unclear. Recently, multiple studies have suggested that there are a lot of commonalities in the pathogenesis of different types of autoimmune diseases. Therefore, the integrative analysis across different autoimmune diseases may reveal more clues about the pathogenesis.

Graves’ disease (GD), rheumatoid arthritis (RA), systemic lupus erythematosus (SLE) and systemic sclerosis (SSc) are all typical autoimmune diseases. They share commonalities of occurring more often in women than in men (Shoenfeld et al., 2008), the production of autoantibodies (Genain et al., 1999; Weetman, 2000; van Boekel et al., 2001; Sherer et al., 2004; Steen, 2005), the triggering of immune abnormalities in CD4+ or CD8+ T cells (Covas et al., 1992; Firestein, 2003; Hussein et al., 2005; McFarland and Martin, 2007; Crispin et al., 2010) and multiple risk loci for the diseases (Kochi, 2016). Thus, research across these autoimmune diseases may reveal similarities in their pathogenesis.

Epigenetics refers to the study of heritable changes in gene function without alterations in DNA sequence (Bird, 2007). A number of environmental risk factors could wield influence over the pathogenesis of autoimmune diseases through epigenetic mechanisms (Zhang and Zhang, 2015), which provide a crucial link between environmental and genetic risk factors of the diseases. DNA methylation is one of the major epigenetic mechanisms, and it plays an important role in determining gene function (Hedrich and Tsokos, 2011). Recently, multiple lines of evidence have shown that dysregulated DNA methylation plays a critical part in the onset of autoimmune diseases, including GD, RA, SLE and SSc (Hedrich and Tsokos, 2011; Cai et al., 2015). Hence, further exploration of the DNA methylation patterns of these diseases can help to elucidate the underlying pathogenesis.

In the present study, we report the integrative analysis of genome-wide DNA methylomes in CD4+ T cells of GD, RA, SLE, SSc patients, as well as in CD8+ T cells of GD, SSc patients. Autoimmune responses mediated by CD4+ and CD8+ T cells play a key role in the diseases mentioned above (O’Garra et al., 1997; Walter and Santamaria, 2005). Commonalities of DNA methylation in these autoimmune diseases were identified. Specific DNA methylation signatures were also evaluated for the potential diagnostic value of these autoimmune diseases.

## Materials and Methods

### Sample and Data Collection

DNA methylation microarray data of GD/RA/SLE patients and corresponding control individuals were acquired from the GEO database [GSE71957 (Limbach et al., 2016), GSE71841 (Guo et al., 2017) and GSE59250 (Absher et al., 2013)], while raw data of SSc patients and matched controls were sourced from our previous study (Ding et al., 2018). All data collected were based on Illumina Methylation 450K array platform. The clinical and demographic information of the patients and healthy subjects is summarized in Supplementary Table 1.

### Preprocessing of Methylation Data

Initial quality control and preprocessing of raw data in the form of IDAT files were conducted in R (R Core Team, 2013) with the Bioconductor package RnBeads (Assenov et al., 2014). The IDAT files were processed separately for each study. Probes whose last three bases of the target sequence overlap with SNP and cross-hybridizing probes whose sequence maps to multiple genomic coordinates were removed before further analysis. The “greedycut” procedure was conducted to filter out the probes with a detection *P*-value > 0.05 in any of the samples. In addition, probes located on sex chromosomes and probes with many missing values were also filtered out. Then, the “noob” method was used to perform background subtraction. Finally, beta-mixture quantile normalization (BMIQ) of the methylation beta values was conducted to adjust the deviation caused by different types of probes (type I and type II).

### Data Integration and Batch Normalization

Preprocessed data of the diseases were adjusted to a unified format. Methylation beta values of samples from multiple disease datasets were then integrated together in both CD4+ and CD8+ sample subsets. Batch normalization was conducted with the SVA package (Leek et al., 2012) to remove batch effect among arrays used for samples of different diseases.

### Differential Methylation Analysis

*P*-values of the probes involved in the integrated methylation data were computed with a linear regression model, including gender, age and ethnicity as covariates. Benjamini and Hochberg false discovery rate (FDR) (Benjamini and Hochberg, 1995) was employed to adjust the raw *P*-values for multiple testing. CpG sites with *P*-values after FDR of < 0.01 were defined as differentially methylated sites (DMS). Further gene ontology (GO) analysis was conducted with g:Profiler (Reimand et al., 2016) using CpGs with the most significant methylation difference (*N* = 50).

### Gene and CpG Island Features of Methylation Sites

Location of the methylation sites in relation to gene and CpG island subregions were annotated based on the annotation files provided by Illumina. Gene-associated features TSS1500, TSS200, 5^′^ UTR and 1st exon were collectively referred to as promoter regions. CpG sites that were located in different parts of these genes were counted separately.

### ANOVA and Clustering Analysis

One-way ANOVA (analysis of variance) was used for multiple comparisons of methylation beta values among patients with different diseases. The analysis was not carried out in CD8+ samples since there were only two patient groups. Hierarchical clustering analysis was performed on the top 50 CpG sites showing the largest variation across all the patient groups based on one-way ANOVA to examine the relatedness among methylation patterns of the diseases. Clustering analysis was also conducted for the most significant DMS (*N* = 50) and DMS at type I IFN-related genes across all samples, respectively. Heat maps were generated to visualize the results of clustering analysis.

### Meta-Analysis

Sample sizes, means and standard deviations of single CpG site’s methylation beta values for patient and control groups were worked out in all the diseases and consolidated together. Meta-analysis was then conducted with the metafor package (Viechtbauer, 2010). The data were pooled and calculated using the DerSimonian and Laird random effects model.

### Binary Prediction Analysis and ROC Evaluation

Binary logistic regression model was selected to fit the ROC (receiver operating characteristic) curve. The area under the ROC curve (AUC) was calculated.

## Results

In order to recognize the commonalities of different autoimmune diseases, including GD, RA, SLE, and SSc, we collected Illumina Methylation 450K microarray data of 116 patients with GD/RA/SLE/SSc and 117 corresponding controls in CD4+ T cells. Similarly, the methylation data of 61 patients with GD/SSc and 55 control individuals in CD8+ T cells were also collected. The data were acquired from public database and our previous work. The detailed clinical characteristics of patients and healthy controls are described in Supplementary Table 1. Stringent quality filtering, exclusion of cross-hybridizing and SNP-containing CpGs, and normalization for batches (as described in Materials and methods) were conducted before further analysis.

### Genome-Wide DNA Methylation Profiling of Autoimmune Diseases

**FIGURE 1:**
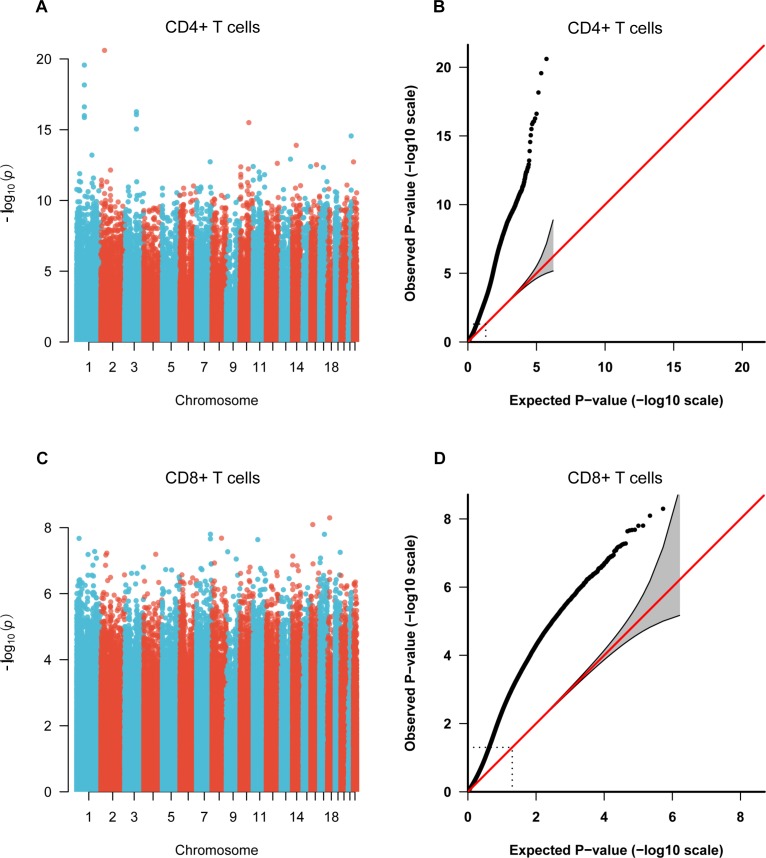
Manhattan plots and Quantile-Quantile (QQ) plots of *P*-values for each CpG site included in the genome-wide methylation analysis between patients and control individuals. **(A,C)** Manhattan plots of the methylation profiles for CD4+ and CD8+ T cells, respectively. X-axis shows the chromosomal position of investigated CpG sites, and y-axis represents the –log10 (*P*-value) for each CpG site. **(B,D)** QQ plots of the methylation profiles for CD4+ and CD8+ T cells, respectively.

To identify the common epigenetic changes associated with the above autoimmune diseases, we performed genome-wide DNA methylation analysis of the patients and controls in both CD4+ and CD8+ T cell subsets. After stringent quality control and differential methylation analysis, we identified 15,289 and 9,295 DMS in CD4+ and CD8+ T cells, respectively (Supplementary Tables 2, 3 and Figure 1). In the CD4+ T cell methylation dataset, 7,889 of these sites were hypomethylated and 7,400 were hypermethylated, with more hypomethylated sites than hypermethylated sites, which is quite different from the pattern observed in cancer (e.g., more hyper- than hypo-methylated sites were observed in gastric cancer and colorectal cancer) (Zouridis et al., 2012; Naumov et al., 2013), while in line with the proportion in GD, RA and SLE (over 50% of the differentially methylated CpGs identified, respectively, were hypomethylated) (Absher et al., 2013; Limbach et al., 2016; Guo et al., 2017); 5,471 and 3,824 probes were hypermethylated and hypomethylated in CD8+ T cells of patients. We also found that DMS were mainly located within genes and outside of CpG islands (Supplementary Figure 1). Over 40% of the DMS were identified within intragenic (body) regions, and more than 30% of the DMS were observed in promoter regions. In relation to CpG islands, only a small proportion (<10%) of all identified DMS were associated with well-defined CpG island regions. In the comparative analysis of two cell subsets’ methylation patterns, we found 5,129 of the DMS were shared between CD4+ and CD8+ T cells, while 10,160 DMS were unique to CD4+ T cells and 4,166 DMS to CD8+ T cells (Supplementary Figure 2). Altogether, we identified 6,027 and 4,358 differentially methylated genes (DMG) in CD4+ and CD8+ T cells, respectively (Table 1), with 2,645 DMG shared between these two subtypes.

**Table 1:** Top 20 differentially methylated genes in CD4+ and CD8+ T cells between patients and controls.

**CD4+ T cells**				**CD8+ T cells**			
**Gene**	**McaM^a^**	**McoM^b^**	**FDR**	**Gene**	**McaM**	**McoM**	**FDR**
*EIF2AK2*	0.22	0.32	9.21 × 10^-16^	*TTC39C*	0.32	0.16	1.07 × 10^-3^
*IFI44L*	0.69	0.81	5.02 × 10^-15^	*ITPRIPL2*	0.86	0.82	1.07 × 10^-3^
*PARP9-DTX3L*	0.54	0.68	3.99 × 10^-12^	*PTDSS1*	0.43	0.28	1.07 × 10^-3^
*IFIT1*	0.83	0.91	1.30 × 10^-11^	*RCAN3*	0.35	0.19	1.07 × 10^-3^
*MX1*	0.6	0.76	9.33 × 10^-11^	*RAPSN*	0.89	0.95	1.07 × 10^-3^
*ETV3*	0.34	0.23	1.78 × 10^-9^	*FAM129A*	0.42	0.27	1.67 × 10^-3^
*UPF3A*	0.35	0.24	3.18 × 10^-9^	*FREM1*	0.67	0.53	1.67 × 10^-3^
*PARP12*	0.66	0.74	4.36 × 10^-9^	*CEACAM18*	0.71	0.66	1.67 × 10^-3^
*USP18*	0.58	0.7	4.36 × 10^-9^	*BCL11A*	0.36	0.26	1.67 × 10^-3^
*OAS1*	0.08	0.11	5.21 × 10^-9^	*CD2*	0.31	0.15	1.67 × 10^-3^
*IRF7*	0.66	0.72	7.39 × 10^-9^	*NRXN1*	0.7	0.8	1.67 × 10^-3^
*C10orf99*	0.46	0.4	9.57 × 10^-9^	*PPP1R3E*	0.55	0.48	1.68 × 10^-3^
*INPP4A*	0.36	0.25	1.10 × 10^-8^	*PIK3R6*	0.72	0.82	1.73 × 10^-3^
*CD5*	0.21	0.12	1.44 × 10^-8^	*NR4A3*	0.36	0.46	1.74 × 10^-3^
*LAPTM5*	0.18	0.11	1.82 × 10^-8^	*SMTNL2*	0.85	0.8	1.86 × 10^-3^
*FXYD2*	0.29	0.21	2.00 × 10^-8^	*DOCK5*	0.89	0.84	1.86 × 10^-3^
*FASLG*	0.3	0.2	3.12 × 10^-8^	*GIMAP7*	0.33	0.18	1.86 × 10^-3^
*TBC1D10C*	0.28	0.18	3.60 × 10^-8^	*METTL9;IGSF6*	0.41	0.28	1.86 × 10^-3^
*RSAD2*	0.09	0.15	3.99 × 10^-8^	*GIMAP1*	0.86	0.79	1.86 × 10^-3^
*TSPAN4*	0.47	0.53	3.99 × 10^-8^	*RNASE1*	0.95	0.92	1.86 × 10^-3^


*^*a*^McaM represents mean methylation level of cases.*

*^*b*^McoM represents mean methylation level of control samples.*

*^*c*^Only annotated genes are shown, and only the site with the most significant *P*-value after FDR is shown if the gene has multiple hits.*

**FIGURE 2:**
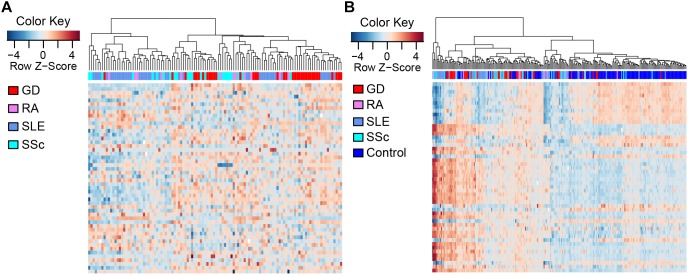
Clustering analysis of CpG sites in CD4+ T cells. Each column represents a sample, each row represents the methylation level of all the samples involved on one CpG site, and the sample clustering tree appears at the top. **(A)** Heat map of CpG sites showing the largest variation (*N* = 50) across CD4+ patient groups. **(B)** Heat map of the top 50 DMS across all CD4+ samples.

To compare the methylation patterns of the autoimmune diseases mentioned above, we preformed hierarchical clustering analysis based on the top 50 CpG sites showing the largest variation across all patient groups (Supplementary Table 4) in CD4+ samples. We found that samples from the four diseases cannot be explicitly distinguished from each other (Figure 2A) (clustering analysis based on the top 50 CpG sites showing the largest variation across all patient and control groups in CD4+ samples is also presented in Supplementary Figure 3). Furthermore, clustering analysis of the top 50 DMS across all samples in CD4+ cells also demonstrated that there is no significant distinction among the methylation signatures of these autoimmune diseases, while different from control individuals (Figure 2B). These results indicate that GD, RA, SLE and SSc patients possess similar methylation patterns, which provided further evidence for the commonalities in the pathogenesis of these diseases.

### Functional Enrichment of the Most Significant Differentially Methylated Genes

**FIGURE 3:**
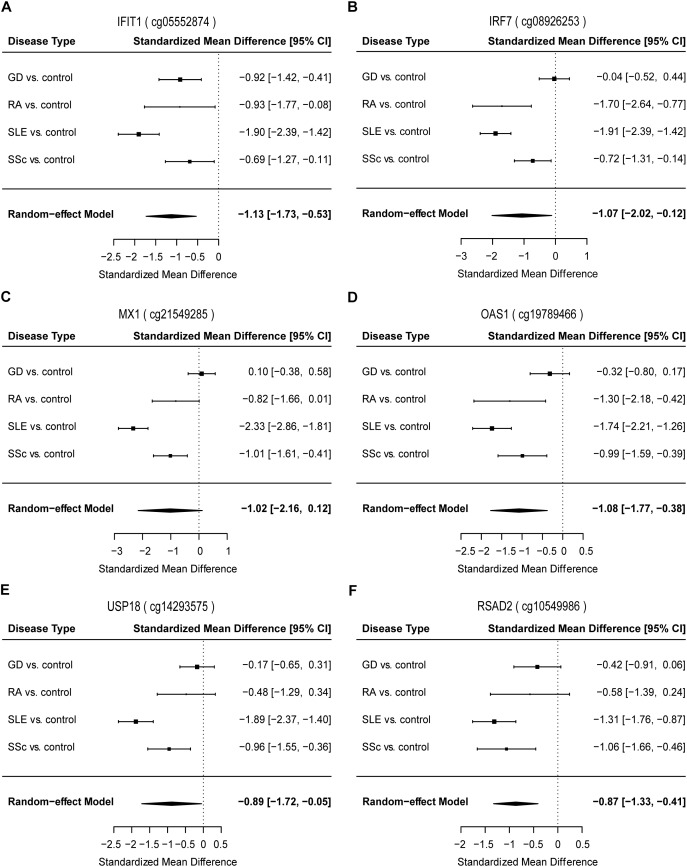
Forest plots of meta-analysis for methylation levels of CpG sites enriched relevant to type I interferon between GD/RA/SLE/SSc patients and control individuals in CD4+ T cell dataset. Disease type, standardized mean difference and 95% CI are labeled. The DerSimonian-Laird estimator was selected to conduct combination estimation for the random-effect model. **(A–F)** Indicate meta-analysis of *IFIT1*, *IRF7*, *MX1*, *OAS1*, *USP18*, and *RSAD2* respectively.

**FIGURE 4:**
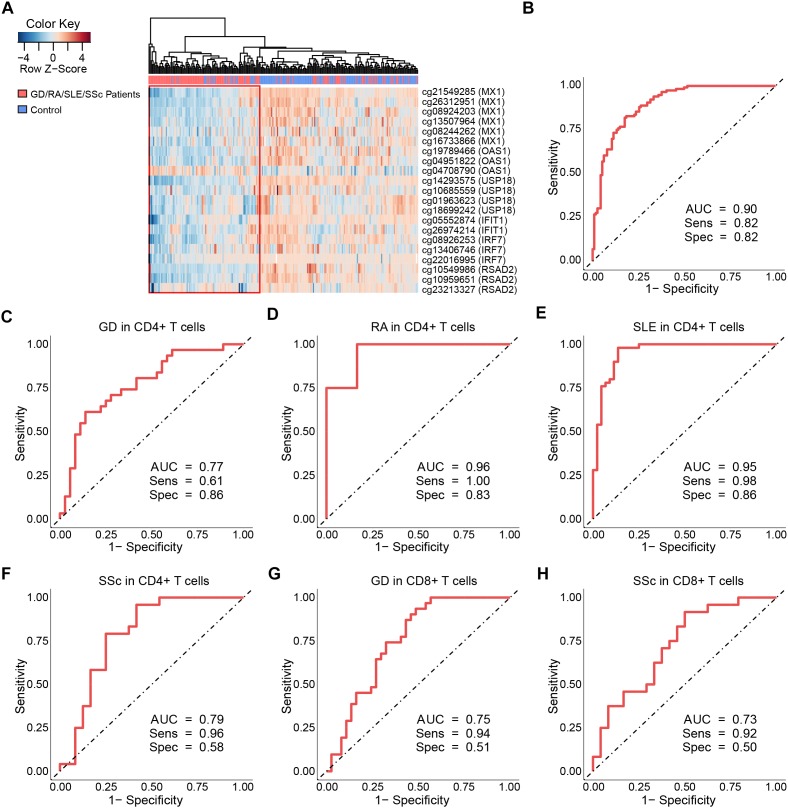
Clustering analysis and ROC curves of the DNA methylation levels at differentially methylated sites on type I interferon-related genes. **(A)** Heat map of the DMS on IFN-related genes annotated by gene ontology (GO) among all samples in CD4+ T cells. Each column represents a sample, each row represents the methylation level of all the samples involved on one CpG site, the sample clustering tree appears at the top. **(B)** ROC curve of DMS found on all IFN-related genes annotated by GO in GD/RA/SLE/SSc patients compared with matched controls in CD4+ T cells. **(C–F)** ROC curves of DMS on *IFI44L* for patients with GD, RA, SLE and SSc in CD4+ T cells, respectively. **(G,H)** ROC curves of DMS on *IFI44L* for patients with GD and SSc in CD8+ T cells, respectively.

To identify common functional characteristics of the DMG, we performed a GO analysis on genes annotated to the most significant DMS (*N* = 50) in each cell type. Interestingly, analysis of CD4+ dataset revealed that the type I interferon signaling pathway, response to type I interferon and cellular response to type I interferon (*P*-value after FDR = 1.98 × 10^-6^) were the most significantly enriched GO terms (Table 2), indicating that type I interferon (IFN) -related genes in CD4+ T cells might play a role in the pathogenesis of autoimmune diseases including GD, RA, SLE and SSc. Specifically, we selected the type I IFN-related genes which differed significantly between diseases and control samples (among top 50 DMS) and also applied meta-analysis simultaneously to further observe the methylation status of these genes when the diseases were pooled together as well as in each disease alone (meta-analyses of all methylation sites from the microarray data were also conducted, with results shown in Supplementary Tables 5, 6). Most of the genes identified (5 out of 6) also presented significant methylation difference between patients and controls. Although we did not observe the differential methylation of each gene in all of the diseases, the overall methylation levels of these genes are significantly lower in GD, RA, SLE and SSc, indicating that hypomethylation signature of type I IFN is shared by different autoimmune diseases (Figure 3 and Supplementary Figure 4). Clustering analysis based on the DMS at type I IFN-related genes showed that most patient samples can be discriminated from controls (Figure 4A). In contrast, functional analysis of CD8+ T cells did not show significant enrichment, and the most enriched GO terms were related to more general biological processes, including regulation of leukocyte activation, regulation of cell adhesion and regulation of T cell activation. Of note, most of the top-ranking terms (6 out of 10) were related to immune system processes (Table 2).

**Table 2:** Gene ontology analysis of genes annotated to differentially methylated sites (top 50) for GD/RA/SLE/SSc CD4+ T cell and GD/SSc CD8+ T cell datasets.

**CD4+ T cells**			**CD8+ T cells**		
**Term**	**FDR**	**Count^a^**	**Term**	**FDR**	**Count**
Type I interferon signaling pathway	1.98 × 10^-6^	6	Regulation of leukocyte activation	0.0667	5
Response to type I interferon	1.98 × 10^-6^	6	Positive regulation of leukocyte apoptotic process	0.0667	2
Cellular response to type I interferon	1.98 × 10^-6^	6	Regulation of cell adhesion	0.0667	6
Negative regulation of viral genome replication	6.76 × 10^-6^	5	Positive regulation of leukocyte cell-cell adhesion	0.0667	4
Defense response to virus	1.01 × 10^-5^	7	Regulation of T cell activation	0.0667	4
Negative regulation of viral life cycle	2.83 × 10^-5^	5	Positive regulation of cell activation	0.0667	5
Regulation of viral genome replication	4.02 × 10^-5^	5	Regulation of leukocyte cell-cell adhesion	0.0667	5
Negative regulation of viral process	4.42 × 10^-5^	5	Postsynaptic membrane organization	0.0667	2
Response to cytokine	4.42 × 10^-5^	11	Leukocyte cell-cell adhesion	0.0667	5
Response to virus	4.63 × 10^-5^	7	Localization within membrane	0.0667	3


*^*a*^Count represents number of genes involved in the GO term.*

### Diagnostic Value of Type I Interferon-Related Genes Annotated by Gene Ontology for GD, RA, SLE, and SSc in CD4+ T Cells

Our study has revealed significant hypomethylation of type I IFN-related genes annotated by GO (*IFIT1*, *IRF7*, *MX1*, *OAS1*, *USP18*, *RSAD2*, Table 2) in CD4+ T cells of GD/RA/SLE/SSc patients compared with controls, and we wanted to further evaluate their ability to distinguish the patients from healthy individuals to see if methylation levels of these genes can be of diagnostic value. Thus, we performed the logistic regression analysis based on the methylation status of type I IFN-related genes in CD4+ T cells. ROC curve analyses were conducted of all the DMS found on type I IFN-related genes between patients and matched controls. The methylation levels of the 21 DMS showed high AUC value of 0.90, with sensitivity and specificity of 0.82 (Figure 4B and Supplementary Table 7). In patients with RA, SSc and SLE (compared with healthy controls), the AUCs were even higher, 1.00, 1.00, and 0.98, respectively, while the AUC in GD patients (compared with healthy controls) was also 0.90 (Supplementary Figures 5A–D and Supplementary Table 7).

To further explore the diagnostic value of each type I IFN-related gene identified, we constructed the ROC curves for DMS found on *IFIT1*, *IRF7*, *MX1*, *OAS1*, *USP18* and *RSAD2*, respectively. In the analysis of *IFIT1*, the AUC value of the 2 DMS in GD/RA/SLE/SSc patients in comparison with healthy controls was 0.82 (sensitivity: 0.84, specificity: 0.71) (Supplementary Figure 6A and Supplementary Table 7). In patients with SLE (compared with control individuals), the AUC was much higher, 0.89; while the AUCs for GD, SSc and RA patients (compared with control individuals) were relatively lower, 0.79, 0.75, and 0.68, respectively (Supplementary Figure 7 and Supplementary Table 7). In the analysis of *IRF7*, AUC value of the 3 DMS in GD/RA/SLE/SSc patients in comparison with healthy controls was 0.78 (sensitivity: 0.74, specificity: 0.75) (Supplementary Figure 6B and Supplementary Table 7). In patients with SLE and RA (compared with control individuals), the AUCs were much higher, 0.92 and 0.88, respectively, while the AUCs for SSc and GD patients (compared with control individuals) were relatively lower, 0.73 and 0.66, respectively (Supplementary Figure 8 and Supplementary Table 7). In the analysis of *MX1*, AUC value of the 6 DMS in GD/RA/SLE/SSc patients in comparison with healthy controls was 0.80 (sensitivity: 0.72, specificity: 0.78) (Supplementary Figure 6C and Supplementary Table 7). In patients with SLE, RA and SSc (compared with control individuals), the AUCs were much higher, 0.92, 0.87, and 0.86, respectively, while the AUC for GD patients (compared with control individuals) was relatively lower, 0.73 (Supplementary Figure 9 and Supplementary Table 7). In the analysis of *OAS1*, the AUC value of the 3 DMS in GD/RA/SLE/SSc patients in comparison with healthy controls was 0.80 (sensitivity: 0.82, specificity: 0.66) (Supplementary Figure 6D and Supplementary Table 7). In patients with SLE, RA and SSc (compared with control individuals), the AUCs were higher, 0.89, 0.84, and 0.82, respectively, while the AUC for GD patients (compared with control individuals) was relatively lower, 0.63 (Supplementary Figure 10 and Supplementary Table 7). In the analysis of *USP18*, AUC value of the 4 DMS in GD/RA/SLE/SSc patients in comparison with healthy controls was 0.78 (sensitivity: 0.87, specificity: 0.62) (Supplementary Figure 6E and Supplementary Table 7). In patients with RA and SLE (compared with control individuals), the AUCs were much higher, 0.92 and 0.89, respectively, while the AUC for SSc and GD patients (compared with control individuals) were the same or relatively lower, 0.78 and 0.62 (Supplementary Figure 11 and Supplementary Table 7). In the analysis of *RSAD2*, AUC value of the 3 DMS in GD/RA/SLE/SSc patients in comparison with healthy controls was 0.81 (sensitivity: 0.83, specificity: 0.69) (Supplementary Figure 6F and Supplementary Table 7). In patients with SLE and RA (compared with control individuals), the AUCs were higher, 0.88 and 0.84, respectively, while the AUCs for SSc and GD patients (compared with control individuals) were relatively lower, 0.77 and 0.75 (Supplementary Figure 12 and Supplementary Table 7).

The results of our analysis imply that DNA methylation levels of type I IFN-related genes identified can be useful biomarkers in the evaluation and diagnosis of GD, RA, SLE, and SSc, and the biomarker panel with all DMS found on the genes involved (*IFIT1*, *IRF7*, *MX1*, *OAS1*, *USP18*, and *RSAD2*) demonstrate the best diagnostic capacity compared to each single type-I IFN related gene, which can correctly discriminate between RA or SSc patients and healthy controls.

### Diagnostic Value of *IFI44L* for GD, RA, SLE, and SSc

Our integrated analysis also found that *IFI44L*, another type I IFN-related gene, is significantly hypomethylated in autoimmune diseases (Supplementary Table 2), although it was not annotated by GO in type I IFN-associated terms. A previous study has identified that the methylation status of *IFI44L* could be utilized as a biomarker for diagnosis of autoimmune disease (Zhao et al., 2016). Therefore, to further assess the diagnostic value of *IFI44L* in GD, RA, SLE and SSc, logistic regression analysis based on the methylation status of *IFI44L* were conducted in CD4+ and CD8+ T cells, respectively. We constructed the ROC curves in both T cell subsets to depict the diagnostic ability of cg06872964 for the above autoimmune diseases, which is the hypomethylated CpG site mentioned in that study (Zhao et al., 2016). The results revealed that the AUC value of the site in GD/RA/SLE/SSc patients in comparison with control individuals for the CD4+ subset was 0.80 (sensitivity: 0.78, specificity: 0.74) (Supplementary Figure 13A and Supplementary Table 8). In patients with SLE (compared with healthy controls), the AUC was much higher, 0.89; while the AUCs for SSc, RA and GD patients (compared with healthy controls) were relatively lower, 0.78, 0.78, and 0.58, respectively (Supplementary Figures 14A–D and Supplementary Table 8). The result for the CD8+ subset showed that the AUC value of the site in GD/SSc patients in comparison with control individuals was 0.65 (sensitivity: 0.93, specificity: 0.34) (Supplementary Figure 13B and Supplementary Table 8). In patients with SSc and GD, the AUCs were 0.73 and 0.61, respectively (Supplementary Figures 14E,F and Supplementary Table 8).

To further assess the diagnostic value of *IFI44L* methylation levels in GD, RA, SLE and SSc, we performed ROC curve analyses of all the DMS found on *IFI44L* between patients and matched controls in CD4+ and CD8+ T cells, respectively. The ROC curve analysis for GD/RA/SLE/SSc patients as compared with healthy controls in CD4+ subset revealed that the methylation levels of the 6 DMS (including cg06872964) had a high AUC value of 0.86, with sensitivity of 0.81 and specificity of 0.80 (Supplementary Figure 15A and Supplementary Table 8). In patients with RA and SLE (compared with control individuals), the AUCs were even higher, 0.96 and 0.95, respectively, while the AUCs for SSc and GD patients (compared with control individuals) were relatively lower, 0.79 and 0.77, respectively (Figure 4C–F). In the ROC curve analysis for GD/SSc patients as compared with healthy controls in the CD8+ subset, the methylation levels of the 2 DMS (*P*-value after FDR of cg06872964 was 0.031, which is higher than our definition of DMS, and thus not included) showed AUC value of 0.75, with sensitivity of 0.89 and specificity of 0.52 (Supplementary Figure 15B and Supplementary Table 8). In patients with GD and SSc (compared with control individuals), the analysis showed AUCs of 0.75 and 0.73, respectively (Figure 4G,H).

The results of our study suggest that aberrant DNA methylation of *IFI44L* is a common signature of autoimmune diseases. GD, RA, SLE, SSc show similar DNA methylation signatures in CD4+ subset; and GD, SSc show similar DNA methylation signatures in CD8+ subset. *IFI44L* methylation may be a promising diagnostic biomarker for GD, RA, SLE and SSc patients, with a biomarker panel including all the DMS found on *IFI44L* between patients and matched controls potentially presenting better diagnostic ability than the single CpG site (cg06872964) reported in the previous research.

## Discussion

In our study, we integrated differential methylation analysis and hierarchical clustering analysis to determine the commonalities across various autoimmune diseases including GD, RA, SLE and SSc in both CD4+ and CD8+ T cells. We found that GD, RA, SLE and SSc patients possess similar DNA methylation signatures. Furthermore, 15,289 and 9,295 DMS were identified between GD/RA/SLE/SSc patients and controls in CD4+ and CD8+ T cells, respectively. Moreover, we identified widespread hypomethylation status of CpG sites located at genes involved in type I IFN signaling in CD4+ T cells, indicating that GD/RA/SLE/SSc patients might be hypersensitive to type I IFN. In addition, methylation status of type I IFN-related genes was revealed as a promising diagnostic biomarker for diagnosis of GD, RA, SLE and SSc.

Aberrant type I IFN function has recently been implicated in several autoimmune diseases, including GD, RA, SLE, and SSc (Bohbot et al., 2006; Chen et al., 2017). A study by Henault et al. (2016) reported that increased amounts of IFN-α derived from self-reactive immunoglobulin E-activated plasmacytoid dendritic cells and the excessive IFN responses triggered were capable of exacerbating self-destructive autoimmune responses in SLE patients. Another study demonstrated that expression of IFN type I regulated genes in RA patients was significantly elevated compared with healthy individuals (van der Pouw Kraan et al., 2007). In addition, more than half of SSc patients showed up-regulation of type I IFN-related genes in both whole blood and peripheral blood mononuclear cells (Tan et al., 2006; York et al., 2007). Moreover, three hepatitis C patients with no known history of familial or personal thyroid disease developed GD after treatment with IFN-α therapy, implicating the strong correlation between type I IFN and GD (Bohbot et al., 2006). Furthermore, multiple type I IFN-related genes were found to be related to the pathogenesis of one or more of the autoimmune diseases mentioned above from genetic association studies, including *STAT4*, *IRF5*, *IFIH1*, and *PLZF* (Stefan et al., 2014; Ghodke-Puranik and Niewold, 2015; Kochi, 2016). In summary, a dysfunctional type I IFN-related pathway is involved in the pathogenesis and progression of GD, RA, SLE, and SSc. Besides, previous published analysis of the methylation dataset for SLE and SSc (Absher et al., 2013; Ding et al., 2018) we adopted in this study also implied hypomethylated genes’ enrichment in type I IFN signaling. In accordance with previous studies, we identified the hypomethylation of type I IFN-related genes annotated by GO, including *IFIT1*, *IRF7*, *MX1*, *OAS1*, *USP18*, and *RSAD2* [which were also identified in SLE before (Absher et al., 2013)] at the epigenetic level in CD4+ T cells of GD/RA/SLE/SSc patients, indicating the aberration of DNA methylation profiles of these genes might play a role in the pathogenesis of the autoimmune diseases in this study.

*IFIT1* is one of the most strongly induced IFN stimulated genes, which preferentially recognizes 2^′^-O unmethylated RNA or uncapped non-self-viral mRNA and inhibits translation initiation or blocks the RNA from the actively replicating pool (Diamond, 2014). An increased expression of *IFIT1* was found in established RA patients, suggesting its potential association of viral infections with autoimmune diseases (Castaneda-Delgado et al., 2017). *IRF7* is a crucial regulator of type I IFN against pathogenic infections, it is predominantly activated by *TLR7* in plasmacytoid dendritic cells with pathogenic nucleic acids recognized by pathogen recognition receptors (Ning et al., 2011). A microarray study revealed up-regulation of *IRF7* mRNA level in peripheral blood cells of SSc patients with early disease (Wu and Assassi, 2013). *MX1* is a key downstream gene of type I IFN and plays a part in mediating the IFN-induced antiviral response against a large variety of viruses (Haller and Kochs, 2011). We and others have reported the up-regulation of *MX1* in SLE and SSc (Coit et al., 2013; Ding et al., 2018). *OAS1* is an IFN-induced antiviral enzyme that can defend against the viral infections by recognizing viral dsRNA (Kristiansen et al., 2011). It is shown to be upregulated in the peripheral blood of SLE patients and overexpressed in SSc skin, compared with normal individuals (Ye et al., 2007; Assassi et al., 2015). USP18 is an IFN-inducible protein, which can deconjugate the ubiquitin-like IFN-stimulated gene 15 (*ISG15*) from target proteins (Potu et al., 2010), and was found to inhibit IFN-induced JAK–STAT signaling at the level of the IFN receptor (Malakhova et al., 2006). Significant overexpression of *USP18* was observed in CD4+ T cells from SLE patients, indicating its role in the pathogenesis of autoimmune diseases (Coit et al., 2013). RSAD2 is also an IFN-inducible protein that inhibits the replication of a broad spectrum of DNA and RNA viruses (Seo et al., 2011). Earlier studies have shown that expression of *RSAD2* is dramatically elevated in patients with RA and SLE, compared to healthy individuals (van der Pouw Kraan et al., 2007; Coit et al., 2013). Previous research has revealed increased expression of *IFIT1*, *IRF7*, *MX1*, *OAS1*, *USP18*, and *RSAD2* in one or more of the four autoimmune diseases, which is consistent with an inverse correlation between gene expression and methylation (Wilson et al., 2007), thus providing further evidence for DNA methylation’s involvement in the regulation of these genes.

Moreover, our study implies that methylation of type I IFN-related genes annotated by GO may be helpful in the evaluation and diagnosis of GD, RA, SLE, and SSc. AUCs of all DMS found on the genes involved were particularly high, and RA and SSc patients could be distinguished from healthy controls via their methylation signatures. Methylation levels of DMS on individual type I IFN-related gene also showed high diagnostic potential, with AUCs all around 0.80.

In addition to the genes mentioned above, *IFI44L* was found to be a hypomethylated type I IFN-related gene in the autoimmune diseases in this work, as well as in primary Sjögren’s syndrome (Imgenberg-Kreuz et al., 2016), although it was not annotated by GO in type I IFN-associated terms. Our analysis suggests that the DNA methylation level of *IFI44L* may facilitate the evaluation and diagnosis of GD, RA, SLE, and SSc, with higher diagnostic ability in CD4+ T cells than CD8+ T cells. AUC, sensitivity and specificity of *IFI44L* methylation (especially with all DMS found on the gene) for RA and SLE compared with matched controls in CD4+ T cells are particularly high, demonstrating that RA and SLE patients can be well discriminated from healthy controls through the methylation differences in *IFI44L*. Our finding for SLE is also in line with a previous biomarker study conducted in SLE peripheral blood (Zhao et al., 2016), which provides further proof for the diagnostic value of *IFI44L*.

## Conclusion

In conclusion, we performed, for the first time, an integrative study of multiple autoimmune diseases’ genome-wide DNA methylation patterns in GD, RA, SLE, and SSc. We identified the similarity of methylation profiles across these diseases and the common hypomethylation signature of type I IFN-related genes in CD4+ T cells of GD/RA/SLE/SSc patients, which may underpin the known association of type I IFN with autoimmune diseases. However, since the Illumina Human Methylation 450K BeadChip covers only about 2% of all the CpG sites in the human genome, we could only quantify the methylation profiles of a limited number of CpG sites presented for each gene. Besides, traditional GWAS/EWAS analysis requires the independence of the variables and which is corrected for SNP-based association, methylation status of CpG sites in the promoter of same gene in methylation 450K are usually highly correlated and it is the most important reason of the relatively strong genomic inflation for Human Methylation 450K array based association study, though uncontrolled confounding factors might also be a driver. Age and gender distribution are also a little different among multiple studies, caused by the disease characteristics, as the male-female ratio for SLE, SSc, GD and RA are 1:6∼10, 1:3∼6, 1:6, 1:3, respectively, etc. Nevertheless, the identification of commonalities among various autoimmune diseases’ DNA methylation patterns will help to facilitate the understanding of the pathogenesis of these diseases, especially the effect of type I IFN, and provide reference for future research. Moreover, potential diagnostic value of type I IFN-related genes were presented in our study, indicating tracking the methylation level of these genes as biomarkers may be useful for monitoring disease progression and response to therapy. However, the data was not separated into training and test data set when carrying out ROC analysis due to limited sample size, which might lead to inflation of AUC. Therefore, with these early stage findings, further exploration in larger sample size will be required to confirm the diagnostic utility of these biomarkers.

## Author Contributions

JW, LJ, and SC contributed to the conception, design and final approval of the submitted version. SC, WP, and SG contributed to the integrative analysis of multiple methylation datasets. DH generated RA dataset for the study and provided suggestion to the idea and data interpretation. SC and SG drafted the manuscript. All authors read and approved the final manuscript.

## Conflict of Interest Statement

The authors declare that the research was conducted in the absence of any commercial or financial relationships that could be construed as a potential conflict of interest.
