# Supplementary material for: Genome-Wide DNA Methylation Profiles Reveal Common Epigenetic Patterns of Interferon-Related Genes in Multiple Autoimmune Diseases
Source: Front Genet. 2019 Apr 5;10:223. doi: 10.3389/fgene.2019.00223 (PMC6459983; doi:10.3389/fgene.2019.00223)
Supplement: Supplementary file 1 [file Table_1.DOCX]

**­­­Supplementary tables**

**Supplementary Table 1. Characteristics of patients and healthy controls**

|  |  | **CD4+ T cells** | | | | | | | | | | |  | **CD8+ T cells** | | | | |
| --- | --- | --- | --- | --- | --- | --- | --- | --- | --- | --- | --- | --- | --- | --- | --- | --- | --- | --- |
|  |  | **GD samples** | |  | **RA samples** | |  | **SLE samples** | |  | **SSc samples** | |  | **GD samples** | |  | **SSc samples** | |
|  |  | **Patients** | **Controls** |  | **Patients** | **Controls** |  | **Patients** | **Controls** |  | **Patients** | **Controls** |  | **Patients** | **Controls** |  | **Patients** | **Controls** |
| **Individuals, n** |  | 36 | 31 |  | 12 | 12 |  | 44 | 50 |  | 24 | 24 |  | 37 | 31 |  | 24 | 24 |
| **Women, n (%)** |  | 32 (89) | 28 (90) |  | 9 (75) | 9 (75) |  | 37 (84) | 39 (78) |  | 17 (71) | 19 (79) |  | 33 (89) | 28 (90) |  | 17 (71) | 19 (79) |
| **Age, years, (mean±SD)** |  | 56.2±15.5 | 53.7±11.9 |  | 40.8±11.5 | 40.6±11.4 |  | 31.0±8.4 | 29.2±6.3 |  | 47.4±14.1 | 46.8±10.0 |  | 55.0±16.3 | 53.7±11.9 |  | 47.4±14.1 | 46.8±10.0 |
| **Ethnicity** |  | Caucasian | Caucasian |  | Chinese | Chinese |  | Caucasian/African | Caucasian /African |  | Chinese | Chinese |  | Caucasian | Caucasian |  | Chinese | Chinese |

**Supplementary Table 2. Top 200 differentially methylated CpG sites in CD4+ T cells between GD/RA/SLE/SSc patients and control individuals**

| **Gene** | **Chr** | **Position** | **Cgname** | **Mean_case** | **Mean_control** | **Diff** | **P-value** | **FDR** | **FC** |
| --- | --- | --- | --- | --- | --- | --- | --- | --- | --- |
| *EIF2AK2* | 2 | 37383568 | cg17326313 | 0.22 | 0.32 | 0.10 | 2.49E-21 | 9.21E-16 | 0.69 |
| *IFI44L* | 1 | 79085586 | cg03607951 | 0.69 | 0.81 | 0.12 | 2.71E-20 | 5.02E-15 | 0.85 |
| *IFI44L* | 1 | 79085713 | cg17980508 | 0.62 | 0.74 | 0.11 | 6.91E-19 | 8.52E-14 | 0.85 |
| *IFI44L* | 1 | 79085765 | cg00855901 | 0.43 | 0.55 | 0.12 | 2.44E-17 | 2.26E-12 | 0.78 |
| *PARP9;DTX3L* | 3 | 122281975 | cg00959259 | 0.54 | 0.68 | 0.15 | 5.40E-17 | 3.99E-12 | 0.79 |
| *PARP9;DTX3L* | 3 | 122281881 | cg22930808 | 0.74 | 0.86 | 0.12 | 8.62E-17 | 5.31E-12 | 0.87 |
| *IFI44L* | 1 | 79085250 | cg06872964 | 0.54 | 0.63 | 0.10 | 1.02E-16 | 5.38E-12 | 0.85 |
| *IFI44L* | 1 | 79088769 | cg05696877 | 0.48 | 0.62 | 0.15 | 1.37E-16 | 6.35E-12 | 0.76 |
| *IFIT1* | 10 | 91153143 | cg05552874 | 0.83 | 0.91 | 0.07 | 3.15E-16 | 1.30E-11 | 0.92 |
| *PARP9;DTX3L* | 3 | 122281939 | cg08122652 | 0.71 | 0.82 | 0.10 | 8.92E-16 | 3.30E-11 | 0.88 |
| *MX1* | 21 | 42799141 | cg21549285 | 0.60 | 0.76 | 0.16 | 2.77E-15 | 9.33E-11 | 0.79 |
|  | 14 | 59066094 | cg02078710 | 0.36 | 0.28 | 0.08 | 1.27E-14 | 3.92E-10 | 1.28 |
| *ETV3* | 1 | 157103641 | cg16374333 | 0.34 | 0.23 | 0.11 | 6.25E-14 | 1.78E-09 | 1.50 |
| *UPF3A* | 13 | 115046279 | cg07218880 | 0.35 | 0.24 | 0.10 | 1.20E-13 | 3.18E-09 | 1.42 |
| *PARP12* | 7 | 139761087 | cg05994974 | 0.66 | 0.74 | 0.08 | 1.86E-13 | 4.36E-09 | 0.89 |
| *USP18* | 22 | 18635460 | cg14293575 | 0.58 | 0.70 | 0.12 | 1.89E-13 | 4.36E-09 | 0.83 |
| *OAS1* | 12 | 113344923 | cg19789466 | 0.08 | 0.11 | 0.03 | 2.40E-13 | 5.21E-09 | 0.72 |
|  | 16 | 57643932 | cg00531137 | 0.20 | 0.12 | 0.08 | 2.99E-13 | 6.15E-09 | 1.70 |
|  | 15 | 99978986 | cg22700246 | 0.37 | 0.30 | 0.08 | 3.98E-13 | 7.39E-09 | 1.26 |
| *IRF7* | 11 | 614761 | cg08926253 | 0.66 | 0.72 | 0.06 | 4.00E-13 | 7.39E-09 | 0.92 |
|  | 10 | 8373659 | cg09234252 | 0.27 | 0.18 | 0.10 | 4.22E-13 | 7.44E-09 | 1.54 |
|  | 17 | 38440015 | cg26854588 | 0.39 | 0.28 | 0.10 | 4.61E-13 | 7.76E-09 | 1.37 |
| *C10orf99* | 10 | 85939451 | cg25526001 | 0.46 | 0.40 | 0.07 | 5.95E-13 | 9.57E-09 | 1.17 |
| *INPP4A* | 2 | 99062999 | cg16469046 | 0.36 | 0.25 | 0.11 | 7.15E-13 | 1.10E-08 | 1.44 |
| *CD5* | 11 | 60869960 | cg24674703 | 0.21 | 0.12 | 0.10 | 9.74E-13 | 1.44E-08 | 1.83 |
| *LAPTM5* | 1 | 31223850 | cg15459165 | 0.18 | 0.11 | 0.06 | 1.28E-12 | 1.82E-08 | 1.53 |
|  | 10 | 8373522 | cg04640972 | 0.27 | 0.17 | 0.09 | 1.43E-12 | 1.96E-08 | 1.53 |
| *FXYD2* | 11 | 117698911 | cg22335223 | 0.29 | 0.21 | 0.07 | 1.51E-12 | 2.00E-08 | 1.34 |
|  | 11 | 69240844 | cg15297628 | 0.31 | 0.24 | 0.08 | 2.18E-12 | 2.78E-08 | 1.33 |
|  | 10 | 8373450 | cg17505469 | 0.35 | 0.24 | 0.11 | 2.30E-12 | 2.83E-08 | 1.44 |
| *FASLG* | 1 | 172628065 | cg06983746 | 0.30 | 0.20 | 0.10 | 2.62E-12 | 3.12E-08 | 1.47 |
| *TBC1D10C* | 11 | 67174843 | cg17161520 | 0.28 | 0.18 | 0.10 | 3.12E-12 | 3.60E-08 | 1.57 |
| *RSAD2* | 2 | 7018153 | cg10549986 | 0.09 | 0.15 | 0.06 | 3.66E-12 | 3.99E-08 | 0.61 |
| *TSPAN4* | 11 | 844085 | cg19303748 | 0.47 | 0.53 | 0.05 | 3.67E-12 | 3.99E-08 | 0.90 |
| *ARID1A* | 1 | 27029290 | cg05649922 | 0.18 | 0.13 | 0.04 | 4.43E-12 | 4.68E-08 | 1.34 |
|  | 17 | 74978687 | cg03728799 | 0.35 | 0.24 | 0.11 | 4.69E-12 | 4.70E-08 | 1.47 |
| *ZNF148* | 3 | 125053815 | cg26313511 | 0.81 | 0.89 | 0.08 | 4.80E-12 | 4.70E-08 | 0.91 |
| *BCL11A* | 2 | 60748951 | cg24361098 | 0.41 | 0.33 | 0.07 | 4.83E-12 | 4.70E-08 | 1.21 |
| *ZNF642* | 1 | 40945066 | cg11654904 | 0.74 | 0.72 | 0.02 | 4.96E-12 | 4.71E-08 | 1.03 |
|  | 10 | 76947956 | cg05021029 | 0.47 | 0.39 | 0.08 | 5.37E-12 | 4.96E-08 | 1.21 |
| *OR10T2* | 1 | 158369112 | cg15593510 | 0.50 | 0.42 | 0.08 | 6.03E-12 | 5.44E-08 | 1.18 |
| *RSAD2* | 2 | 7018020 | cg10959651 | 0.24 | 0.30 | 0.06 | 7.21E-12 | 6.35E-08 | 0.79 |
|  | 10 | 8373416 | cg27470208 | 0.37 | 0.26 | 0.11 | 8.00E-12 | 6.88E-08 | 1.44 |
| *LIME1* | 20 | 62367805 | cg06653796 | 0.11 | 0.06 | 0.05 | 8.21E-12 | 6.90E-08 | 1.91 |
| *CDC42BPB* | 14 | 103412980 | cg21057323 | 0.25 | 0.20 | 0.06 | 9.00E-12 | 7.40E-08 | 1.29 |
| *HECA* | 6 | 139483193 | cg23280720 | 0.19 | 0.10 | 0.08 | 9.47E-12 | 7.57E-08 | 1.81 |
|  | 10 | 90031426 | cg09221159 | 0.21 | 0.13 | 0.08 | 9.62E-12 | 7.57E-08 | 1.66 |
| *CMPK2* | 2 | 7004578 | cg01028142 | 0.88 | 0.93 | 0.05 | 1.01E-11 | 7.82E-08 | 0.95 |
| *FRMD4A* | 10 | 13831250 | cg08791347 | 0.38 | 0.31 | 0.07 | 1.11E-11 | 7.86E-08 | 1.24 |
| *FASLG* | 1 | 172628263 | cg00071250 | 0.30 | 0.21 | 0.09 | 1.11E-11 | 7.86E-08 | 1.42 |
| *STK17A* | 7 | 43652643 | cg11022926 | 0.38 | 0.29 | 0.09 | 1.12E-11 | 7.86E-08 | 1.32 |
| *CX3CR1* | 3 | 39309355 | cg03341377 | 0.42 | 0.34 | 0.07 | 1.12E-11 | 7.86E-08 | 1.21 |
|  | 7 | 142494244 | cg16767506 | 0.39 | 0.30 | 0.09 | 1.14E-11 | 7.86E-08 | 1.30 |
| *MPZL3* | 11 | 118100782 | cg25155064 | 0.27 | 0.17 | 0.10 | 1.15E-11 | 7.86E-08 | 1.58 |
| *ABHD12B* | 14 | 51342320 | cg21052932 | 0.18 | 0.09 | 0.09 | 1.35E-11 | 8.82E-08 | 1.93 |
|  | 5 | 969899 | cg07406498 | 0.46 | 0.41 | 0.05 | 1.36E-11 | 8.82E-08 | 1.14 |
| *ZFAND3* | 6 | 37826480 | cg04752885 | 0.62 | 0.55 | 0.07 | 1.36E-11 | 8.82E-08 | 1.12 |
| *ACTB* | 7 | 5567862 | cg01741041 | 0.17 | 0.11 | 0.05 | 1.42E-11 | 9.04E-08 | 1.45 |
| *CCM2* | 7 | 45075791 | cg12573289 | 0.32 | 0.27 | 0.06 | 1.44E-11 | 9.05E-08 | 1.21 |
| *KIAA1310* | 2 | 97260774 | cg12927730 | 0.29 | 0.22 | 0.07 | 1.51E-11 | 9.30E-08 | 1.30 |
| *C18orf1* | 18 | 13267090 | cg21737421 | 0.47 | 0.38 | 0.09 | 1.54E-11 | 9.36E-08 | 1.23 |
|  | 1 | 1093940 | cg07213830 | 0.63 | 0.67 | 0.05 | 1.66E-11 | 9.87E-08 | 0.93 |
|  | 13 | 42615991 | cg25365958 | 0.58 | 0.66 | 0.07 | 1.72E-11 | 9.99E-08 | 0.89 |
| *RCAN3* | 1 | 24861604 | cg20146241 | 0.12 | 0.07 | 0.05 | 1.73E-11 | 9.99E-08 | 1.71 |
|  | 17 | 48857353 | cg15046489 | 0.40 | 0.30 | 0.10 | 1.80E-11 | 1.02E-07 | 1.33 |
| *MX1* | 21 | 42797847 | cg26312951 | 0.22 | 0.29 | 0.08 | 1.85E-11 | 1.04E-07 | 0.74 |
| *LCK* | 1 | 32716961 | cg05350315 | 0.21 | 0.12 | 0.09 | 2.04E-11 | 1.11E-07 | 1.73 |
| *RCAN3* | 1 | 24861818 | cg01519464 | 0.35 | 0.23 | 0.12 | 2.05E-11 | 1.11E-07 | 1.51 |
| *SNAP47* | 1 | 227954102 | cg22356061 | 0.14 | 0.08 | 0.05 | 2.09E-11 | 1.12E-07 | 1.62 |
| *ZMYND17* | 10 | 75193254 | cg08945443 | 0.56 | 0.48 | 0.08 | 2.14E-11 | 1.13E-07 | 1.16 |
| *CX3CR1* | 3 | 39309435 | cg24310395 | 0.28 | 0.19 | 0.10 | 2.20E-11 | 1.14E-07 | 1.52 |
| *CD2* | 1 | 117296954 | cg03016153 | 0.27 | 0.17 | 0.10 | 2.39E-11 | 1.21E-07 | 1.62 |
| *SCRN1* | 7 | 30009236 | cg04858110 | 0.57 | 0.52 | 0.06 | 2.40E-11 | 1.21E-07 | 1.11 |
| *SBNO2* | 19 | 1155030 | cg19649900 | 0.73 | 0.82 | 0.09 | 2.42E-11 | 1.21E-07 | 0.89 |
| *RPS6KA2* | 6 | 166825479 | cg06491415 | 0.38 | 0.28 | 0.09 | 2.58E-11 | 1.27E-07 | 1.33 |
| *KLF2* | 19 | 16437789 | cg02668248 | 0.24 | 0.16 | 0.08 | 2.82E-11 | 1.37E-07 | 1.50 |
| *MIR145;LOC728264* | 5 | 148810180 | cg22941668 | 0.87 | 0.92 | 0.05 | 2.87E-11 | 1.38E-07 | 0.94 |
|  | 22 | 50984368 | cg18770635 | 0.89 | 0.92 | 0.03 | 2.90E-11 | 1.38E-07 | 0.96 |
| *GIMAP7* | 7 | 150217056 | cg16532400 | 0.36 | 0.25 | 0.11 | 3.04E-11 | 1.42E-07 | 1.47 |
| *CD5* | 11 | 60869969 | cg00299736 | 0.11 | 0.07 | 0.04 | 3.15E-11 | 1.46E-07 | 1.54 |
| *TNK2* | 3 | 195632915 | cg15065340 | 0.55 | 0.65 | 0.10 | 3.23E-11 | 1.46E-07 | 0.85 |
|  | 2 | 37418009 | cg05627557 | 0.85 | 0.90 | 0.05 | 3.25E-11 | 1.46E-07 | 0.94 |
| *SESN1* | 6 | 109341365 | cg17117243 | 0.33 | 0.23 | 0.09 | 3.36E-11 | 1.50E-07 | 1.41 |
| *FAM53B* | 10 | 126428818 | cg26517376 | 0.34 | 0.28 | 0.06 | 3.48E-11 | 1.53E-07 | 1.22 |
|  | 6 | 42739639 | cg05945608 | 0.12 | 0.08 | 0.04 | 3.57E-11 | 1.55E-07 | 1.55 |
| *IFIT5* | 10 | 91173811 | cg06376949 | 0.56 | 0.63 | 0.07 | 3.81E-11 | 1.64E-07 | 0.89 |
|  | 5 | 969918 | cg13674369 | 0.52 | 0.46 | 0.06 | 4.15E-11 | 1.75E-07 | 1.12 |
| *F12* | 5 | 176836695 | cg06625767 | 0.84 | 0.90 | 0.05 | 4.16E-11 | 1.75E-07 | 0.94 |
|  | 3 | 156848003 | cg14969094 | 0.17 | 0.11 | 0.05 | 4.24E-11 | 1.76E-07 | 1.48 |
| *PTDSS1* | 8 | 97296348 | cg22062539 | 0.48 | 0.39 | 0.09 | 4.41E-11 | 1.79E-07 | 1.23 |
| *COPG* | 3 | 128995479 | cg12216435 | 0.19 | 0.13 | 0.07 | 4.42E-11 | 1.79E-07 | 1.53 |
| *ZAP70* | 2 | 98329337 | cg25095518 | 0.17 | 0.10 | 0.07 | 4.45E-11 | 1.79E-07 | 1.64 |
|  | 6 | 474629 | cg11682724 | 0.14 | 0.09 | 0.05 | 4.51E-11 | 1.79E-07 | 1.57 |
| *PPM1M* | 3 | 52280329 | cg07773769 | 0.18 | 0.13 | 0.05 | 4.63E-11 | 1.82E-07 | 1.39 |
| *ZFP36L1* | 14 | 69256690 | cg06617636 | 0.34 | 0.24 | 0.10 | 4.76E-11 | 1.85E-07 | 1.39 |
| *RNF166* | 16 | 88770966 | cg03776194 | 0.18 | 0.13 | 0.06 | 4.92E-11 | 1.90E-07 | 1.44 |
|  | 1 | 85764021 | cg27183818 | 0.40 | 0.31 | 0.09 | 5.03E-11 | 1.90E-07 | 1.29 |
| *FREM1* | 9 | 14749903 | cg14506515 | 0.59 | 0.51 | 0.08 | 5.03E-11 | 1.90E-07 | 1.17 |
| *RPTOR* | 17 | 78764256 | cg16115689 | 0.24 | 0.18 | 0.06 | 5.31E-11 | 1.98E-07 | 1.33 |
| *KLF2* | 19 | 16437362 | cg18473733 | 0.35 | 0.28 | 0.07 | 5.36E-11 | 1.98E-07 | 1.24 |
| *GATA3* | 10 | 8103415 | cg25630514 | 0.27 | 0.17 | 0.10 | 5.73E-11 | 2.07E-07 | 1.58 |
| *LY9* | 1 | 160765919 | cg18865207 | 0.11 | 0.06 | 0.05 | 5.75E-11 | 2.07E-07 | 1.73 |
|  | 1 | 111213827 | cg17516825 | 0.44 | 0.35 | 0.09 | 5.77E-11 | 2.07E-07 | 1.25 |
| *CBFA2T3* | 16 | 89009929 | cg08843248 | 0.37 | 0.33 | 0.04 | 6.09E-11 | 2.16E-07 | 1.12 |
|  | 12 | 122444580 | cg14480046 | 0.31 | 0.21 | 0.11 | 6.22E-11 | 2.17E-07 | 1.51 |
| *PRR5L* | 11 | 36397123 | cg26601310 | 0.19 | 0.12 | 0.07 | 6.23E-11 | 2.17E-07 | 1.55 |
| *FAM49B* | 8 | 130898833 | cg19123356 | 0.33 | 0.27 | 0.06 | 6.27E-11 | 2.17E-07 | 1.21 |
| *LIME1* | 20 | 62367893 | cg20513976 | 0.14 | 0.08 | 0.07 | 6.41E-11 | 2.19E-07 | 1.86 |
| *EHD4* | 15 | 42227210 | cg22451412 | 0.11 | 0.07 | 0.03 | 7.15E-11 | 2.41E-07 | 1.49 |
| *TC2N* | 14 | 92334029 | cg08796342 | 0.23 | 0.15 | 0.09 | 7.18E-11 | 2.41E-07 | 1.58 |
| *LY9* | 1 | 160765805 | cg18920397 | 0.25 | 0.17 | 0.08 | 7.28E-11 | 2.43E-07 | 1.48 |
| *SEZ6L* | 22 | 26776311 | cg12636835 | 0.39 | 0.30 | 0.08 | 7.35E-11 | 2.43E-07 | 1.27 |
| *SMARCA4* | 19 | 11074303 | cg08315613 | 0.29 | 0.21 | 0.08 | 7.42E-11 | 2.43E-07 | 1.36 |
| *LCP1* | 13 | 46744500 | cg07880943 | 0.44 | 0.38 | 0.06 | 7.57E-11 | 2.46E-07 | 1.15 |
| *PRKCQ* | 10 | 6618723 | cg26818464 | 0.34 | 0.25 | 0.08 | 7.86E-11 | 2.51E-07 | 1.33 |
| *BIN3* | 8 | 22497061 | cg15942979 | 0.28 | 0.18 | 0.10 | 7.86E-11 | 2.51E-07 | 1.52 |
| *OR5AU1* | 14 | 21624639 | cg07555731 | 0.29 | 0.21 | 0.08 | 8.17E-11 | 2.56E-07 | 1.39 |
| *FNBP1* | 9 | 132652466 | cg10531986 | 0.53 | 0.47 | 0.07 | 8.24E-11 | 2.56E-07 | 1.14 |
| *NR1H3* | 11 | 47276469 | cg24909660 | 0.73 | 0.80 | 0.06 | 8.27E-11 | 2.56E-07 | 0.92 |
| *PARP14* | 3 | 122400474 | cg01948202 | 0.14 | 0.21 | 0.07 | 8.32E-11 | 2.56E-07 | 0.68 |
| *PTK2B* | 8 | 27219512 | cg12743031 | 0.26 | 0.17 | 0.09 | 8.88E-11 | 2.72E-07 | 1.54 |
| *LIME1* | 20 | 62368256 | cg12413156 | 0.23 | 0.15 | 0.08 | 9.29E-11 | 2.82E-07 | 1.53 |
| *RPS6KA2* | 6 | 166825084 | cg08109681 | 0.37 | 0.26 | 0.11 | 9.43E-11 | 2.84E-07 | 1.43 |
| *MGAT4A* | 2 | 99280070 | cg13584531 | 0.30 | 0.19 | 0.11 | 9.78E-11 | 2.91E-07 | 1.56 |
| *TTC7A* | 2 | 47171414 | cg05927789 | 0.23 | 0.17 | 0.07 | 9.90E-11 | 2.91E-07 | 1.39 |
| *THEMIS* | 6 | 128222378 | cg02098313 | 0.21 | 0.13 | 0.08 | 9.90E-11 | 2.91E-07 | 1.62 |
| *ABLIM1* | 10 | 116301354 | cg17056676 | 0.28 | 0.21 | 0.08 | 1.01E-10 | 2.93E-07 | 1.36 |
| *PWWP2B* | 10 | 134211874 | cg25303150 | 0.51 | 0.54 | 0.04 | 1.02E-10 | 2.95E-07 | 0.93 |
| *SMARCA4* | 19 | 11074428 | cg22898082 | 0.29 | 0.20 | 0.09 | 1.13E-10 | 3.24E-07 | 1.45 |
| *CORO1A;LOC606724* | 16 | 30198509 | cg06749872 | 0.37 | 0.31 | 0.06 | 1.14E-10 | 3.25E-07 | 1.19 |
| *UCP2* | 11 | 73692155 | cg25429672 | 0.27 | 0.18 | 0.09 | 1.18E-10 | 3.30E-07 | 1.50 |
| *LAX1* | 1 | 203734505 | cg12022621 | 0.20 | 0.13 | 0.07 | 1.19E-10 | 3.30E-07 | 1.49 |
| *MYOM2* | 8 | 2079369 | cg08004620 | 0.20 | 0.12 | 0.08 | 1.19E-10 | 3.30E-07 | 1.63 |
| *LAMP3* | 3 | 182876556 | cg18030943 | 0.43 | 0.37 | 0.06 | 1.23E-10 | 3.36E-07 | 1.16 |
| *TNFRSF1B* | 1 | 12238546 | cg15526535 | 0.36 | 0.28 | 0.08 | 1.24E-10 | 3.36E-07 | 1.28 |
| *LAPTM5* | 1 | 31217240 | cg19510565 | 0.24 | 0.15 | 0.09 | 1.24E-10 | 3.36E-07 | 1.61 |
| *POFUT2* | 21 | 46686908 | cg01267908 | 0.54 | 0.49 | 0.05 | 1.25E-10 | 3.36E-07 | 1.09 |
| *DCLK3* | 3 | 36782467 | cg21113478 | 0.34 | 0.29 | 0.06 | 1.25E-10 | 3.36E-07 | 1.20 |
|  | 2 | 177356020 | cg07484739 | 0.36 | 0.29 | 0.07 | 1.28E-10 | 3.42E-07 | 1.23 |
| *MICAL1* | 6 | 109777592 | cg13206063 | 0.83 | 0.88 | 0.05 | 1.31E-10 | 3.47E-07 | 0.95 |
| *ZNF853* | 7 | 6659785 | cg06987246 | 0.22 | 0.15 | 0.06 | 1.34E-10 | 3.52E-07 | 1.42 |
| *MIR145;LOC728264* | 5 | 148810177 | cg11671363 | 0.85 | 0.91 | 0.06 | 1.38E-10 | 3.54E-07 | 0.93 |
| *ANKFY1* | 17 | 4079652 | cg00277591 | 0.29 | 0.18 | 0.10 | 1.39E-10 | 3.54E-07 | 1.57 |
| *PRDX5* | 11 | 64087106 | cg10718809 | 0.76 | 0.83 | 0.06 | 1.39E-10 | 3.54E-07 | 0.92 |
| *IL2RB* | 22 | 37546220 | cg21307484 | 0.19 | 0.13 | 0.06 | 1.39E-10 | 3.54E-07 | 1.47 |
| *TDRD7* | 9 | 100175029 | cg11516606 | 0.10 | 0.13 | 0.02 | 1.40E-10 | 3.54E-07 | 0.81 |
| *CTNNA3* | 10 | 69372655 | cg01249544 | 0.37 | 0.30 | 0.07 | 1.44E-10 | 3.62E-07 | 1.22 |
| *CLEC2D* | 12 | 9822287 | cg12810837 | 0.23 | 0.13 | 0.10 | 1.47E-10 | 3.67E-07 | 1.74 |
| *CMPK2* | 2 | 7006627 | cg24935042 | 0.13 | 0.17 | 0.04 | 1.49E-10 | 3.69E-07 | 0.76 |
|  | 12 | 19557334 | cg20894640 | 0.46 | 0.37 | 0.09 | 1.52E-10 | 3.72E-07 | 1.25 |
| *ODF3B* | 22 | 50971140 | cg20098015 | 0.62 | 0.68 | 0.06 | 1.52E-10 | 3.72E-07 | 0.91 |
|  | 2 | 48787750 | cg18984200 | 0.59 | 0.56 | 0.03 | 1.63E-10 | 3.94E-07 | 1.06 |
| *CSNK1G3* | 5 | 122851781 | cg27054084 | 0.26 | 0.18 | 0.08 | 1.64E-10 | 3.94E-07 | 1.46 |
| *43352* | 17 | 75315108 | cg07863022 | 0.78 | 0.84 | 0.06 | 1.64E-10 | 3.94E-07 | 0.93 |
| *ZNF609* | 15 | 64944023 | cg08462055 | 0.19 | 0.12 | 0.07 | 1.66E-10 | 3.96E-07 | 1.55 |
| *PLEKHA1* | 10 | 124181965 | cg02556345 | 0.21 | 0.15 | 0.06 | 1.67E-10 | 3.96E-07 | 1.41 |
| *RABGAP1L* | 1 | 174843909 | cg05702218 | 0.51 | 0.57 | 0.06 | 1.68E-10 | 3.96E-07 | 0.90 |
| *PLCG1* | 20 | 39767358 | cg07893801 | 0.23 | 0.14 | 0.09 | 1.72E-10 | 4.04E-07 | 1.62 |
| *C10orf105;CDH23* | 10 | 73486801 | cg13740185 | 0.18 | 0.12 | 0.06 | 1.76E-10 | 4.09E-07 | 1.50 |
|  | 1 | 111179008 | cg07706695 | 0.20 | 0.11 | 0.09 | 1.77E-10 | 4.09E-07 | 1.87 |
|  | 8 | 20350779 | cg10145533 | 0.27 | 0.16 | 0.11 | 1.81E-10 | 4.15E-07 | 1.68 |
|  | 2 | 42123725 | cg05075562 | 0.41 | 0.33 | 0.08 | 1.85E-10 | 4.21E-07 | 1.23 |
|  | 11 | 75943228 | cg24520862 | 0.24 | 0.16 | 0.08 | 1.86E-10 | 4.22E-07 | 1.47 |
| *FLJ23834* | 7 | 105662809 | cg02366772 | 0.11 | 0.07 | 0.05 | 1.90E-10 | 4.26E-07 | 1.74 |
|  | 17 | 61042898 | cg04522575 | 0.21 | 0.13 | 0.08 | 1.90E-10 | 4.26E-07 | 1.56 |
| *FKBP5* | 6 | 35693573 | cg23416081 | 0.47 | 0.55 | 0.08 | 1.97E-10 | 4.36E-07 | 0.86 |
|  | 6 | 37521698 | cg08766762 | 0.48 | 0.37 | 0.11 | 1.97E-10 | 4.36E-07 | 1.28 |
| *LBH* | 2 | 30457110 | cg17250262 | 0.22 | 0.15 | 0.07 | 2.01E-10 | 4.40E-07 | 1.48 |
| *FAM53B* | 10 | 126315761 | cg24916358 | 0.09 | 0.06 | 0.04 | 2.02E-10 | 4.40E-07 | 1.60 |
|  | 14 | 31679612 | cg09834503 | 0.62 | 0.54 | 0.08 | 2.02E-10 | 4.40E-07 | 1.15 |
| *MX1* | 21 | 42798747 | cg08924203 | 0.14 | 0.20 | 0.06 | 2.06E-10 | 4.45E-07 | 0.72 |
| *EVL* | 14 | 100532036 | cg17813891 | 0.24 | 0.14 | 0.10 | 2.10E-10 | 4.51E-07 | 1.67 |
| *PRF1* | 10 | 72362694 | cg23059461 | 0.17 | 0.13 | 0.04 | 2.14E-10 | 4.58E-07 | 1.32 |
|  | 10 | 85951128 | cg15227994 | 0.16 | 0.11 | 0.05 | 2.16E-10 | 4.58E-07 | 1.50 |
| *LAX1* | 1 | 203734396 | cg17711527 | 0.15 | 0.09 | 0.06 | 2.17E-10 | 4.59E-07 | 1.61 |
| *RPTOR* | 17 | 78800774 | cg16638092 | 0.81 | 0.87 | 0.05 | 2.22E-10 | 4.62E-07 | 0.94 |
| *EIF3B* | 7 | 2419958 | cg24695071 | 0.90 | 0.93 | 0.03 | 2.22E-10 | 4.62E-07 | 0.97 |
|  | 14 | 91695017 | cg13152690 | 0.27 | 0.19 | 0.08 | 2.22E-10 | 4.62E-07 | 1.43 |
|  | 21 | 45576085 | cg01772743 | 0.32 | 0.24 | 0.08 | 2.25E-10 | 4.66E-07 | 1.34 |
| *SETD1A* | 16 | 30976186 | cg10020520 | 0.84 | 0.90 | 0.06 | 2.27E-10 | 4.67E-07 | 0.94 |
| *CIRH1A* | 16 | 69200251 | cg08327038 | 0.36 | 0.28 | 0.09 | 2.31E-10 | 4.71E-07 | 1.31 |
| *IKZF3* | 17 | 37956902 | cg08441850 | 0.30 | 0.21 | 0.09 | 2.34E-10 | 4.73E-07 | 1.40 |
| *EPC1* | 10 | 32621403 | cg07092111 | 0.35 | 0.29 | 0.07 | 2.34E-10 | 4.73E-07 | 1.24 |
| *GIMAP7* | 7 | 150216489 | cg03800150 | 0.34 | 0.24 | 0.10 | 2.36E-10 | 4.74E-07 | 1.42 |
| *MX1* | 21 | 42792703 | cg13507964 | 0.10 | 0.12 | 0.02 | 2.37E-10 | 4.74E-07 | 0.82 |
| *LTB* | 6 | 31550090 | cg19279042 | 0.17 | 0.12 | 0.05 | 2.39E-10 | 4.75E-07 | 1.40 |
| *HIF1A* | 14 | 62166083 | cg23174662 | 0.40 | 0.33 | 0.07 | 2.42E-10 | 4.76E-07 | 1.22 |
|  | 5 | 1316264 | cg26209169 | 0.19 | 0.12 | 0.07 | 2.42E-10 | 4.76E-07 | 1.61 |
| *RPTOR* | 17 | 78800806 | cg08939850 | 0.77 | 0.83 | 0.06 | 2.47E-10 | 4.84E-07 | 0.92 |
| *RANBP9* | 6 | 13695743 | cg08622666 | 0.36 | 0.26 | 0.10 | 2.50E-10 | 4.86E-07 | 1.36 |
|  | 7 | 155790512 | cg13104880 | 0.38 | 0.31 | 0.07 | 2.51E-10 | 4.86E-07 | 1.23 |
| *PA2G4;ERBB3* | 12 | 56497073 | cg10056728 | 0.37 | 0.29 | 0.08 | 2.53E-10 | 4.86E-07 | 1.27 |
| *DOCK10* | 2 | 225639708 | cg13742400 | 0.79 | 0.86 | 0.07 | 2.55E-10 | 4.86E-07 | 0.92 |
|  | 21 | 35575070 | cg19118951 | 0.68 | 0.74 | 0.06 | 2.55E-10 | 4.86E-07 | 0.92 |
| *FASLG* | 1 | 172628020 | cg10161121 | 0.28 | 0.18 | 0.10 | 2.58E-10 | 4.88E-07 | 1.59 |
| *CREG2* | 2 | 101966951 | cg06333233 | 0.53 | 0.46 | 0.07 | 2.59E-10 | 4.88E-07 | 1.15 |
|  | 2 | 40147801 | cg01644640 | 0.43 | 0.35 | 0.08 | 2.61E-10 | 4.90E-07 | 1.23 |
| *KRTAP12-4;C21orf29* | 21 | 46074169 | cg07273342 | 0.43 | 0.36 | 0.07 | 2.68E-10 | 5.01E-07 | 1.19 |
| *BZRAP1* | 17 | 56401800 | cg11645674 | 0.51 | 0.58 | 0.08 | 2.72E-10 | 5.02E-07 | 0.87 |
| *CSK* | 15 | 75077691 | cg01735277 | 0.20 | 0.12 | 0.09 | 2.74E-10 | 5.02E-07 | 1.75 |

**Supplementary Table 3. Top 200 differentially methylated CpG sites in CD8+ T cells between GD/SSc patients and control individuals**

| **Gene** | **Chr** | **Position** | **Cgname** | **Mean_case** | **Mean_control** | **Diff** | **P-value** | **FDR** | **FC** |
| --- | --- | --- | --- | --- | --- | --- | --- | --- | --- |
| *TTC39C* | 18 | 21572748 | cg17205313 | 0.32 | 0.16 | 0.16 | 5.05E-09 | 1.07E-03 | 2.03 |
| *ITPRIPL2* | 16 | 19127172 | cg18596043 | 0.86 | 0.82 | 0.04 | 8.08E-09 | 1.07E-03 | 1.05 |
|  | 7 | 142494244 | cg16767506 | 0.35 | 0.24 | 0.11 | 1.58E-08 | 1.07E-03 | 1.49 |
|  | 17 | 48857353 | cg15046489 | 0.39 | 0.24 | 0.15 | 1.59E-08 | 1.07E-03 | 1.65 |
| *PTDSS1* | 8 | 97296348 | cg22062539 | 0.43 | 0.28 | 0.15 | 2.09E-08 | 1.07E-03 | 1.56 |
| *RCAN3* | 1 | 24861818 | cg01519464 | 0.35 | 0.19 | 0.16 | 2.12E-08 | 1.07E-03 | 1.86 |
|  | 7 | 142494213 | cg13264840 | 0.41 | 0.23 | 0.18 | 2.18E-08 | 1.07E-03 | 1.80 |
| *RAPSN* | 11 | 47471339 | cg13047308 | 0.89 | 0.95 | 0.06 | 2.31E-08 | 1.07E-03 | 0.93 |
| *FAM129A* | 1 | 184944785 | cg12141056 | 0.42 | 0.27 | 0.15 | 5.28E-08 | 1.67E-03 | 1.57 |
| *FREM1* | 9 | 14749903 | cg14506515 | 0.67 | 0.53 | 0.13 | 5.38E-08 | 1.67E-03 | 1.25 |
| *CEACAM18* | 19 | 51984816 | cg03110722 | 0.71 | 0.66 | 0.05 | 5.58E-08 | 1.67E-03 | 1.08 |
| *BCL11A* | 2 | 60748951 | cg24361098 | 0.36 | 0.26 | 0.10 | 5.92E-08 | 1.67E-03 | 1.37 |
|  | 4 | 122706839 | cg16509531 | 0.86 | 0.81 | 0.06 | 6.42E-08 | 1.67E-03 | 1.07 |
| *CD2* | 1 | 117296954 | cg03016153 | 0.31 | 0.15 | 0.16 | 6.50E-08 | 1.67E-03 | 2.10 |
| *NRXN1* | 2 | 51074939 | cg13204432 | 0.70 | 0.80 | 0.10 | 6.75E-08 | 1.67E-03 | 0.88 |
| *PPP1R3E* | 14 | 23772616 | cg23620184 | 0.55 | 0.48 | 0.07 | 7.27E-08 | 1.68E-03 | 1.14 |
| *PIK3R6* | 17 | 8762014 | cg00409104 | 0.72 | 0.82 | 0.10 | 8.35E-08 | 1.73E-03 | 0.88 |
|  | 1 | 203002593 | cg20414082 | 0.84 | 0.78 | 0.06 | 8.42E-08 | 1.73E-03 | 1.08 |
| *NR4A3* | 9 | 102588232 | cg13655635 | 0.36 | 0.46 | 0.10 | 8.94E-08 | 1.74E-03 | 0.79 |
| *SMTNL2* | 17 | 4500399 | cg14257656 | 0.85 | 0.80 | 0.05 | 1.15E-07 | 1.86E-03 | 1.06 |
| *DOCK5* | 8 | 25229721 | cg24449706 | 0.89 | 0.84 | 0.05 | 1.18E-07 | 1.86E-03 | 1.06 |
| *GIMAP7* | 7 | 150216489 | cg03800150 | 0.33 | 0.18 | 0.15 | 1.25E-07 | 1.86E-03 | 1.81 |
| *METTL9;IGSF6* | 16 | 21658497 | cg06257110 | 0.41 | 0.28 | 0.13 | 1.25E-07 | 1.86E-03 | 1.48 |
| *GIMAP1* | 7 | 150413164 | cg24116513 | 0.86 | 0.79 | 0.06 | 1.26E-07 | 1.86E-03 | 1.08 |
| *RNASE1* | 14 | 21271313 | cg13718960 | 0.95 | 0.92 | 0.02 | 1.34E-07 | 1.86E-03 | 1.03 |
| *CD247* | 1 | 167486978 | cg14278300 | 0.38 | 0.25 | 0.13 | 1.37E-07 | 1.86E-03 | 1.55 |
|  | 2 | 47915862 | cg10211062 | 0.70 | 0.65 | 0.05 | 1.38E-07 | 1.86E-03 | 1.08 |
|  | 14 | 69281297 | cg04718050 | 0.18 | 0.11 | 0.06 | 1.43E-07 | 1.86E-03 | 1.56 |
| *LRRC15* | 3 | 194089473 | cg14180696 | 0.38 | 0.31 | 0.07 | 1.57E-07 | 1.86E-03 | 1.22 |
| *MGEA5* | 10 | 103574626 | cg02505177 | 0.29 | 0.19 | 0.10 | 1.58E-07 | 1.86E-03 | 1.52 |
|  | 11 | 75943228 | cg24520862 | 0.23 | 0.13 | 0.10 | 1.71E-07 | 1.86E-03 | 1.75 |
|  | 7 | 44906917 | cg19663284 | 0.68 | 0.60 | 0.08 | 1.73E-07 | 1.86E-03 | 1.13 |
| *AMACR* | 5 | 33997484 | cg18397975 | 0.92 | 0.96 | 0.05 | 1.86E-07 | 1.86E-03 | 0.95 |
| *VOPP1* | 7 | 55637719 | cg21959598 | 0.32 | 0.18 | 0.14 | 1.88E-07 | 1.86E-03 | 1.81 |
|  | 5 | 95514729 | cg00283187 | 0.72 | 0.65 | 0.07 | 1.92E-07 | 1.86E-03 | 1.10 |
| *SYTL2* | 11 | 85460604 | cg11691844 | 0.80 | 0.89 | 0.09 | 1.96E-07 | 1.86E-03 | 0.90 |
| *TSHR* | 14 | 81426234 | cg14373410 | 0.45 | 0.29 | 0.16 | 2.02E-07 | 1.86E-03 | 1.54 |
|  | 10 | 90031426 | cg09221159 | 0.23 | 0.11 | 0.12 | 2.19E-07 | 1.86E-03 | 2.04 |
|  | 12 | 106539009 | cg21703255 | 0.75 | 0.68 | 0.07 | 2.21E-07 | 1.86E-03 | 1.10 |
| *TTC39C* | 18 | 21572634 | cg05401069 | 0.65 | 0.52 | 0.13 | 2.23E-07 | 1.86E-03 | 1.26 |
|  | 16 | 79306292 | cg27027055 | 0.31 | 0.17 | 0.14 | 2.38E-07 | 1.86E-03 | 1.85 |
| *EIF4E3* | 3 | 71779016 | cg22888463 | 0.11 | 0.06 | 0.05 | 2.42E-07 | 1.86E-03 | 1.86 |
| *CALML3* | 10 | 5567123 | cg26178664 | 0.92 | 0.89 | 0.03 | 2.43E-07 | 1.86E-03 | 1.03 |
| *KIAA1257* | 3 | 128710390 | cg25752703 | 0.79 | 0.89 | 0.10 | 2.43E-07 | 1.86E-03 | 0.89 |
| *TMPRSS4* | 11 | 117957665 | cg01728830 | 0.90 | 0.85 | 0.05 | 2.51E-07 | 1.86E-03 | 1.06 |
| *CDKAL1* | 6 | 20689061 | cg20488123 | 0.25 | 0.13 | 0.11 | 2.69E-07 | 1.86E-03 | 1.83 |
| *SFRS16* | 19 | 45567180 | cg26985354 | 0.78 | 0.85 | 0.06 | 2.70E-07 | 1.86E-03 | 0.92 |
| *PDCD1* | 2 | 242799553 | cg10994870 | 0.81 | 0.76 | 0.05 | 2.70E-07 | 1.86E-03 | 1.07 |
| *ETV3* | 1 | 157103641 | cg16374333 | 0.31 | 0.16 | 0.14 | 2.76E-07 | 1.86E-03 | 1.88 |
| *AKNA* | 9 | 117156883 | cg13999433 | 0.82 | 0.90 | 0.08 | 2.76E-07 | 1.86E-03 | 0.91 |
| *VANGL1* | 1 | 116191657 | cg25773695 | 0.22 | 0.13 | 0.09 | 2.77E-07 | 1.86E-03 | 1.71 |
| *COASY* | 17 | 40713676 | cg24535823 | 0.87 | 0.82 | 0.05 | 2.79E-07 | 1.86E-03 | 1.06 |
| *SIRPG* | 20 | 1639816 | cg13408605 | 0.51 | 0.39 | 0.12 | 2.82E-07 | 1.86E-03 | 1.30 |
|  | 12 | 112200694 | cg18714086 | 0.80 | 0.73 | 0.07 | 2.86E-07 | 1.86E-03 | 1.10 |
|  | 1 | 172608644 | cg17756730 | 0.26 | 0.16 | 0.11 | 2.93E-07 | 1.86E-03 | 1.69 |
| *ERGIC1* | 5 | 172280684 | cg07496207 | 0.83 | 0.91 | 0.08 | 3.10E-07 | 1.86E-03 | 0.92 |
| *INPP4A* | 2 | 99062999 | cg16469046 | 0.31 | 0.18 | 0.13 | 3.21E-07 | 1.86E-03 | 1.74 |
| *SAMD3* | 6 | 130544832 | cg16493584 | 0.40 | 0.24 | 0.16 | 3.33E-07 | 1.86E-03 | 1.67 |
|  | 13 | 101241225 | cg27248148 | 0.83 | 0.79 | 0.04 | 3.51E-07 | 1.86E-03 | 1.05 |
| *MED24* | 17 | 38175389 | cg15412772 | 0.83 | 0.89 | 0.06 | 3.57E-07 | 1.86E-03 | 0.94 |
| *GRK6* | 5 | 176861823 | cg02523893 | 0.88 | 0.84 | 0.04 | 3.58E-07 | 1.86E-03 | 1.05 |
| *ANKFY1* | 17 | 4079652 | cg00277591 | 0.28 | 0.15 | 0.13 | 3.61E-07 | 1.86E-03 | 1.85 |
| *NR1H3* | 11 | 47276469 | cg24909660 | 0.75 | 0.84 | 0.10 | 3.67E-07 | 1.86E-03 | 0.89 |
|  | 16 | 72516874 | cg09699830 | 0.45 | 0.33 | 0.12 | 3.72E-07 | 1.86E-03 | 1.36 |
| *TTC39C* | 18 | 21572656 | cg18719665 | 0.47 | 0.33 | 0.13 | 3.74E-07 | 1.86E-03 | 1.40 |
| *SMARCA4* | 19 | 11074303 | cg08315613 | 0.25 | 0.15 | 0.10 | 3.78E-07 | 1.86E-03 | 1.63 |
| *LCK* | 1 | 32717002 | cg17078393 | 0.47 | 0.32 | 0.15 | 3.79E-07 | 1.86E-03 | 1.46 |
| *GIMAP7* | 7 | 150217081 | cg01260502 | 0.58 | 0.45 | 0.13 | 3.90E-07 | 1.86E-03 | 1.29 |
| *ETS1* | 11 | 128395450 | cg03295554 | 0.24 | 0.13 | 0.11 | 3.92E-07 | 1.86E-03 | 1.80 |
| *GRHL3* | 1 | 24648203 | cg06376426 | 0.21 | 0.15 | 0.06 | 3.99E-07 | 1.86E-03 | 1.37 |
| *MYOF* | 10 | 95218729 | cg27220117 | 0.78 | 0.73 | 0.06 | 3.99E-07 | 1.86E-03 | 1.08 |
| *AAK1* | 2 | 69793701 | cg17101703 | 0.21 | 0.13 | 0.08 | 4.00E-07 | 1.86E-03 | 1.61 |
| *43352* | 17 | 75276069 | cg01733438 | 0.77 | 0.84 | 0.07 | 4.02E-07 | 1.86E-03 | 0.92 |
|  | 6 | 170525514 | cg01345395 | 0.86 | 0.82 | 0.04 | 4.06E-07 | 1.86E-03 | 1.05 |
| *DOCK5* | 8 | 25045589 | cg24805985 | 0.89 | 0.84 | 0.05 | 4.14E-07 | 1.86E-03 | 1.06 |
| *ZNF804B;MGC26647* | 7 | 88425148 | cg22946147 | 0.91 | 0.87 | 0.03 | 4.19E-07 | 1.86E-03 | 1.04 |
| *SH3TC1* | 4 | 8230689 | cg07248017 | 0.40 | 0.29 | 0.11 | 4.19E-07 | 1.86E-03 | 1.39 |
|  | 11 | 123351497 | cg05868564 | 0.48 | 0.57 | 0.09 | 4.19E-07 | 1.86E-03 | 0.85 |
|  | 10 | 101297508 | cg13424029 | 0.63 | 0.71 | 0.08 | 4.39E-07 | 1.86E-03 | 0.88 |
| *FARS2* | 6 | 5282702 | cg26704043 | 0.73 | 0.81 | 0.08 | 4.40E-07 | 1.86E-03 | 0.90 |
| *TRIM27* | 6 | 28887836 | cg05216056 | 0.90 | 0.96 | 0.06 | 4.41E-07 | 1.86E-03 | 0.94 |
| *SLC16A8* | 22 | 38474147 | cg11324504 | 0.63 | 0.71 | 0.08 | 4.45E-07 | 1.86E-03 | 0.89 |
| *TRIM2* | 4 | 154126481 | cg02832697 | 0.33 | 0.28 | 0.05 | 4.57E-07 | 1.86E-03 | 1.18 |
| *AIM1* | 6 | 106988121 | cg02331198 | 0.80 | 0.89 | 0.09 | 4.61E-07 | 1.86E-03 | 0.90 |
|  | 1 | 56797691 | cg19825277 | 0.83 | 0.76 | 0.07 | 4.66E-07 | 1.86E-03 | 1.09 |
| *HIVEP3* | 1 | 42380309 | cg11735008 | 0.28 | 0.15 | 0.13 | 4.79E-07 | 1.86E-03 | 1.83 |
| *SEZ6L* | 22 | 26776311 | cg12636835 | 0.29 | 0.16 | 0.13 | 4.84E-07 | 1.86E-03 | 1.81 |
| *RCAN3* | 1 | 24861604 | cg20146241 | 0.15 | 0.08 | 0.08 | 4.85E-07 | 1.86E-03 | 1.99 |
| *SBNO2* | 19 | 1155184 | cg10819238 | 0.67 | 0.79 | 0.12 | 4.86E-07 | 1.86E-03 | 0.85 |
| *DNAH2* | 17 | 7701546 | cg06935856 | 0.87 | 0.84 | 0.02 | 4.88E-07 | 1.86E-03 | 1.03 |
| *FLJ45079* | 17 | 75880542 | cg04837616 | 0.84 | 0.91 | 0.07 | 4.91E-07 | 1.86E-03 | 0.93 |
| *GATA3* | 10 | 8103415 | cg25630514 | 0.25 | 0.13 | 0.12 | 4.94E-07 | 1.86E-03 | 1.93 |
| *ELMO1* | 7 | 37024552 | cg08044454 | 0.75 | 0.85 | 0.10 | 4.94E-07 | 1.86E-03 | 0.89 |
| *ERI1* | 8 | 8863572 | cg24386894 | 0.91 | 0.95 | 0.05 | 4.95E-07 | 1.86E-03 | 0.95 |
| *PTCRA* | 6 | 42883624 | cg01928820 | 0.42 | 0.37 | 0.05 | 4.98E-07 | 1.86E-03 | 1.15 |
| *TIGIT* | 3 | 114012659 | cg19421218 | 0.57 | 0.44 | 0.13 | 5.08E-07 | 1.86E-03 | 1.31 |
| *RPS6KA2* | 6 | 166825479 | cg06491415 | 0.35 | 0.23 | 0.12 | 5.13E-07 | 1.86E-03 | 1.50 |
| *MAFK* | 7 | 1575621 | cg10389644 | 0.75 | 0.83 | 0.08 | 5.18E-07 | 1.86E-03 | 0.90 |
| *IKZF3* | 17 | 37956902 | cg08441850 | 0.28 | 0.17 | 0.12 | 5.22E-07 | 1.86E-03 | 1.70 |
|  | 14 | 22974951 | cg02315513 | 0.35 | 0.21 | 0.15 | 5.26E-07 | 1.86E-03 | 1.70 |
| *ADORA2A* | 22 | 24823389 | cg01373166 | 0.36 | 0.26 | 0.10 | 5.27E-07 | 1.86E-03 | 1.37 |
| *IQSEC1* | 3 | 13005914 | cg13071069 | 0.73 | 0.81 | 0.09 | 5.42E-07 | 1.86E-03 | 0.89 |
| *GRK6* | 5 | 176859356 | cg00636527 | 0.26 | 0.17 | 0.09 | 5.44E-07 | 1.86E-03 | 1.53 |
| *PIK3CG* | 7 | 106511741 | cg00661777 | 0.29 | 0.20 | 0.09 | 5.52E-07 | 1.86E-03 | 1.43 |
| *ITK* | 5 | 156608118 | cg09453312 | 0.27 | 0.11 | 0.16 | 5.56E-07 | 1.86E-03 | 2.37 |
| *LMO4* | 1 | 87793607 | cg20633321 | 0.53 | 0.61 | 0.07 | 5.56E-07 | 1.86E-03 | 0.88 |
|  | 14 | 91695017 | cg13152690 | 0.26 | 0.15 | 0.11 | 5.66E-07 | 1.86E-03 | 1.71 |
| *PDGFRB* | 5 | 149510737 | cg02414981 | 0.88 | 0.85 | 0.03 | 5.74E-07 | 1.86E-03 | 1.04 |
| *LIME1* | 20 | 62367998 | cg14396214 | 0.08 | 0.05 | 0.03 | 5.74E-07 | 1.86E-03 | 1.51 |
| *MTMR9L* | 1 | 32708234 | cg16985778 | 0.44 | 0.54 | 0.10 | 5.77E-07 | 1.86E-03 | 0.81 |
| *FOXK1* | 7 | 4746837 | cg23587176 | 0.90 | 0.95 | 0.05 | 5.77E-07 | 1.86E-03 | 0.95 |
| *FASLG* | 1 | 172628263 | cg00071250 | 0.31 | 0.20 | 0.11 | 5.77E-07 | 1.86E-03 | 1.56 |
| *IL2RB* | 22 | 37546325 | cg04995291 | 0.30 | 0.18 | 0.11 | 5.79E-07 | 1.86E-03 | 1.62 |
| *AGTRAP* | 1 | 11795897 | cg08425760 | 0.77 | 0.85 | 0.08 | 5.80E-07 | 1.86E-03 | 0.90 |
| *CDC25B* | 20 | 3778655 | cg14165142 | 0.23 | 0.12 | 0.11 | 5.83E-07 | 1.86E-03 | 1.95 |
| *ITGB7* | 12 | 53599806 | cg26689077 | 0.15 | 0.09 | 0.06 | 5.83E-07 | 1.86E-03 | 1.64 |
|  | 7 | 38316654 | cg26576544 | 0.60 | 0.48 | 0.12 | 5.89E-07 | 1.86E-03 | 1.25 |
| *CMIP* | 16 | 81491312 | cg06627532 | 0.72 | 0.79 | 0.07 | 6.07E-07 | 1.89E-03 | 0.91 |
|  | 11 | 331179 | cg04537282 | 0.35 | 0.24 | 0.11 | 6.09E-07 | 1.89E-03 | 1.45 |
| *RAD51L1* | 14 | 68749962 | cg18825221 | 0.16 | 0.07 | 0.09 | 6.13E-07 | 1.89E-03 | 2.15 |
| *CTSZ* | 20 | 57583195 | cg09315134 | 0.64 | 0.72 | 0.08 | 6.30E-07 | 1.91E-03 | 0.89 |
| *STAT5B* | 17 | 40424590 | cg07402310 | 0.22 | 0.13 | 0.09 | 6.35E-07 | 1.91E-03 | 1.70 |
| *NFE2* | 12 | 54688344 | cg23384027 | 0.79 | 0.85 | 0.06 | 6.37E-07 | 1.91E-03 | 0.93 |
| *43352* | 17 | 75316480 | cg20305489 | 0.92 | 0.95 | 0.03 | 6.40E-07 | 1.91E-03 | 0.97 |
| *PTPRN2* | 7 | 158061805 | cg20673767 | 0.74 | 0.69 | 0.05 | 6.52E-07 | 1.93E-03 | 1.07 |
|  | 15 | 69760994 | cg18477949 | 0.28 | 0.17 | 0.10 | 6.60E-07 | 1.94E-03 | 1.59 |
| *PVT1* | 8 | 128994030 | cg20516032 | 0.30 | 0.16 | 0.13 | 6.85E-07 | 1.96E-03 | 1.83 |
| *LOC647979* | 20 | 34639686 | cg08587504 | 0.84 | 0.89 | 0.05 | 6.93E-07 | 1.96E-03 | 0.94 |
| *CD300C* | 17 | 72542758 | cg10372525 | 0.91 | 0.88 | 0.03 | 7.01E-07 | 1.96E-03 | 1.03 |
|  | 8 | 26276541 | cg22022716 | 0.91 | 0.96 | 0.05 | 7.07E-07 | 1.96E-03 | 0.95 |
| *NOTCH1* | 9 | 139426285 | cg09073701 | 0.76 | 0.83 | 0.07 | 7.08E-07 | 1.96E-03 | 0.92 |
| *LPCAT1* | 5 | 1475096 | cg07516556 | 0.80 | 0.88 | 0.08 | 7.09E-07 | 1.96E-03 | 0.91 |
| *FYCO1* | 3 | 46035439 | cg18682028 | 0.48 | 0.34 | 0.14 | 7.23E-07 | 1.96E-03 | 1.42 |
| *SSBP3* | 1 | 54842404 | cg09417011 | 0.64 | 0.51 | 0.13 | 7.34E-07 | 1.96E-03 | 1.26 |
| *SMARCA4* | 19 | 11074428 | cg22898082 | 0.25 | 0.15 | 0.10 | 7.34E-07 | 1.96E-03 | 1.68 |
| *SORL1* | 11 | 121349740 | cg01657758 | 0.77 | 0.84 | 0.06 | 7.36E-07 | 1.96E-03 | 0.92 |
| *GRK6* | 5 | 176868293 | cg16426715 | 0.81 | 0.77 | 0.04 | 7.46E-07 | 1.96E-03 | 1.05 |
| *SLC35E3* | 12 | 69143431 | cg25194194 | 0.82 | 0.89 | 0.07 | 7.48E-07 | 1.96E-03 | 0.92 |
|  | 11 | 94988642 | cg20062681 | 0.93 | 0.96 | 0.03 | 7.49E-07 | 1.96E-03 | 0.97 |
| *HLA-F* | 6 | 29692399 | cg05358170 | 0.80 | 0.73 | 0.07 | 7.50E-07 | 1.96E-03 | 1.10 |
| *TOX* | 8 | 60020513 | cg06779553 | 0.33 | 0.24 | 0.09 | 7.50E-07 | 1.96E-03 | 1.38 |
|  | 14 | 22925659 | cg08234149 | 0.77 | 0.62 | 0.14 | 7.54E-07 | 1.96E-03 | 1.23 |
|  | 13 | 41860793 | cg09909069 | 0.37 | 0.22 | 0.15 | 7.61E-07 | 1.96E-03 | 1.72 |
| *PRF1* | 10 | 72362730 | cg12433559 | 0.27 | 0.17 | 0.10 | 7.64E-07 | 1.96E-03 | 1.57 |
| *PITPNC1* | 17 | 65471461 | cg15756507 | 0.23 | 0.13 | 0.10 | 7.67E-07 | 1.96E-03 | 1.74 |
|  | 7 | 41919892 | cg02420480 | 0.76 | 0.84 | 0.08 | 7.77E-07 | 1.97E-03 | 0.91 |
| *PDE2A* | 11 | 72380070 | cg23149454 | 0.57 | 0.49 | 0.08 | 7.91E-07 | 1.99E-03 | 1.16 |
| *CUX1* | 7 | 101465705 | cg14519777 | 0.89 | 0.93 | 0.04 | 7.95E-07 | 1.99E-03 | 0.95 |
| *IL2RB* | 22 | 37546220 | cg21307484 | 0.16 | 0.09 | 0.07 | 8.35E-07 | 2.05E-03 | 1.76 |
| *C19orf28* | 19 | 3553032 | cg19584038 | 0.71 | 0.79 | 0.08 | 8.36E-07 | 2.05E-03 | 0.90 |
| *FAM105B* | 5 | 14686255 | cg14717752 | 0.32 | 0.23 | 0.09 | 8.46E-07 | 2.05E-03 | 1.39 |
| *DLC1* | 8 | 13078829 | cg13007701 | 0.84 | 0.90 | 0.06 | 8.48E-07 | 2.05E-03 | 0.94 |
| *TTC39C* | 18 | 21572622 | cg12639429 | 0.64 | 0.52 | 0.12 | 8.74E-07 | 2.05E-03 | 1.22 |
| *SLC12A4* | 16 | 68000763 | cg09086087 | 0.75 | 0.83 | 0.08 | 8.74E-07 | 2.05E-03 | 0.90 |
| *XIRP1* | 3 | 39230030 | cg25574691 | 0.86 | 0.83 | 0.02 | 8.76E-07 | 2.05E-03 | 1.03 |
| *PIK3R1* | 5 | 67583627 | cg09101894 | 0.64 | 0.52 | 0.12 | 8.79E-07 | 2.05E-03 | 1.22 |
| *FAM49B* | 8 | 130898833 | cg19123356 | 0.30 | 0.22 | 0.08 | 8.81E-07 | 2.05E-03 | 1.38 |
|  | 1 | 112135849 | cg16108580 | 0.27 | 0.16 | 0.11 | 8.85E-07 | 2.05E-03 | 1.73 |
| *VGLL4;ATG7* | 3 | 11597941 | cg00668519 | 0.71 | 0.81 | 0.10 | 8.85E-07 | 2.05E-03 | 0.88 |
| *GIMAP4* | 7 | 150264311 | cg13662290 | 0.25 | 0.16 | 0.10 | 8.93E-07 | 2.05E-03 | 1.62 |
| *RAD51L3* | 17 | 33426885 | cg08154963 | 0.85 | 0.93 | 0.08 | 9.01E-07 | 2.05E-03 | 0.91 |
| *FASLG* | 1 | 172628020 | cg10161121 | 0.28 | 0.14 | 0.14 | 9.08E-07 | 2.05E-03 | 1.98 |
| *ITGB2* | 21 | 46332181 | cg24815934 | 0.09 | 0.05 | 0.04 | 9.14E-07 | 2.05E-03 | 1.69 |
| *BRE* | 2 | 28497669 | cg05057534 | 0.78 | 0.87 | 0.09 | 9.18E-07 | 2.05E-03 | 0.90 |
|  | 14 | 103545835 | cg04528445 | 0.71 | 0.67 | 0.05 | 9.19E-07 | 2.05E-03 | 1.07 |
| *LCK* | 1 | 32715428 | cg07571745 | 0.21 | 0.12 | 0.09 | 9.26E-07 | 2.05E-03 | 1.70 |
| *DNTT* | 10 | 98064175 | cg20728490 | 0.24 | 0.18 | 0.06 | 9.27E-07 | 2.05E-03 | 1.34 |
| *SUMO3* | 21 | 46235052 | cg10871721 | 0.77 | 0.86 | 0.09 | 9.37E-07 | 2.05E-03 | 0.90 |
| *SRP68* | 17 | 74067713 | cg07166266 | 0.31 | 0.19 | 0.12 | 9.43E-07 | 2.05E-03 | 1.65 |
| *ROPN1L* | 5 | 10445523 | cg11080651 | 0.34 | 0.40 | 0.06 | 9.51E-07 | 2.05E-03 | 0.86 |
|  | 19 | 45344725 | cg13375295 | 0.38 | 0.28 | 0.10 | 9.57E-07 | 2.05E-03 | 1.38 |
| *LY9* | 1 | 160765805 | cg18920397 | 0.22 | 0.12 | 0.10 | 9.60E-07 | 2.05E-03 | 1.79 |
| *HIP1* | 7 | 75194698 | cg25444339 | 0.79 | 0.88 | 0.09 | 9.67E-07 | 2.05E-03 | 0.90 |
| *HIVEP3* | 1 | 42334284 | cg21830221 | 0.31 | 0.19 | 0.12 | 9.70E-07 | 2.05E-03 | 1.64 |
| *SHANK1* | 19 | 51220273 | cg22626858 | 0.78 | 0.75 | 0.03 | 9.75E-07 | 2.05E-03 | 1.04 |
| *DHX9* | 1 | 182810711 | cg26546113 | 0.41 | 0.29 | 0.13 | 9.88E-07 | 2.05E-03 | 1.44 |
| *ARHGEF16* | 1 | 3382565 | cg15015996 | 0.56 | 0.51 | 0.05 | 9.90E-07 | 2.05E-03 | 1.10 |
| *HLA-E* | 6 | 30460548 | cg09569347 | 0.34 | 0.26 | 0.08 | 9.98E-07 | 2.05E-03 | 1.32 |
|  | 6 | 170525426 | cg04450052 | 0.38 | 0.31 | 0.08 | 1.00E-06 | 2.05E-03 | 1.25 |
|  | 10 | 134818842 | cg21346589 | 0.97 | 0.96 | 0.01 | 1.00E-06 | 2.05E-03 | 1.01 |
| *TBC1D1* | 4 | 37891376 | cg04710179 | 0.87 | 0.92 | 0.04 | 1.01E-06 | 2.05E-03 | 0.95 |
| *GALK2* | 15 | 49462125 | cg25860737 | 0.35 | 0.27 | 0.09 | 1.02E-06 | 2.05E-03 | 1.32 |
| *LIME1* | 20 | 62368256 | cg12413156 | 0.22 | 0.12 | 0.10 | 1.03E-06 | 2.05E-03 | 1.83 |
| *DNM3* | 1 | 172239092 | cg17591816 | 0.88 | 0.93 | 0.05 | 1.03E-06 | 2.05E-03 | 0.95 |
| *SLC6A3* | 5 | 1395203 | cg13557594 | 0.90 | 0.87 | 0.03 | 1.04E-06 | 2.05E-03 | 1.04 |
| *ANKRD13A* | 12 | 110449223 | cg02580045 | 0.78 | 0.72 | 0.06 | 1.04E-06 | 2.05E-03 | 1.08 |
|  | 14 | 101908830 | cg11874976 | 0.33 | 0.21 | 0.12 | 1.04E-06 | 2.05E-03 | 1.55 |
|  | 2 | 20565699 | cg25420254 | 0.72 | 0.66 | 0.06 | 1.05E-06 | 2.05E-03 | 1.09 |
|  | 2 | 58797233 | cg10416784 | 0.32 | 0.20 | 0.12 | 1.06E-06 | 2.05E-03 | 1.63 |
| *FBXO21* | 12 | 117627223 | cg01817067 | 0.91 | 0.86 | 0.04 | 1.06E-06 | 2.05E-03 | 1.05 |
| *GIMAP4* | 7 | 150264987 | cg00323915 | 0.27 | 0.14 | 0.13 | 1.06E-06 | 2.05E-03 | 2.00 |
| *DOCK5* | 8 | 25229610 | cg00914787 | 0.87 | 0.81 | 0.05 | 1.07E-06 | 2.05E-03 | 1.07 |
| *ANKRD11* | 16 | 89461734 | cg02226192 | 0.80 | 0.87 | 0.08 | 1.07E-06 | 2.05E-03 | 0.91 |
| *TBC1D16* | 17 | 77967529 | cg03837680 | 0.60 | 0.68 | 0.07 | 1.08E-06 | 2.05E-03 | 0.89 |
| *YWHAZ* | 8 | 101960390 | cg26192520 | 0.32 | 0.22 | 0.10 | 1.08E-06 | 2.05E-03 | 1.42 |
| *TMC8* | 17 | 76130305 | cg18437480 | 0.22 | 0.15 | 0.07 | 1.08E-06 | 2.05E-03 | 1.47 |
| *NADSYN1* | 11 | 71189490 | cg05785753 | 0.82 | 0.89 | 0.07 | 1.10E-06 | 2.05E-03 | 0.92 |
| *PDE2A* | 11 | 72379867 | cg00239835 | 0.23 | 0.16 | 0.07 | 1.10E-06 | 2.05E-03 | 1.43 |
|  | 1 | 39301897 | cg04094548 | 0.95 | 0.97 | 0.02 | 1.11E-06 | 2.05E-03 | 0.98 |
| *C1orf105* | 1 | 172410217 | cg00501765 | 0.85 | 0.92 | 0.07 | 1.11E-06 | 2.05E-03 | 0.93 |

**Supplementary Table 4. CpG sites showing the largest variation (N=50) across GD, RA, SLE and SSc patients based on ANOVA in CD4+ T cells**

| **Cgname** | **P-value** | **Chr** | **Position** | **Gene** |
| --- | --- | --- | --- | --- |
| cg07839457 | 3.77E-05 | 16 | 57023022 | *NLRC5* |
| cg06188083 | 1.46E-04 | 10 | 91093005 | *IFIT3* |
| cg02478369 | 1.94E-04 | 17 | 15083645 |  |
| cg09084391 | 4.05E-04 | 5 | 346247 | *AHRR* |
| cg21549285 | 5.26E-04 | 21 | 42799141 | *MX1* |
| cg16200531 | 5.63E-04 | 2 | 135532566 |  |
| cg26312951 | 8.42E-04 | 21 | 42797847 | *MX1* |
| cg17511968 | 1.45E-03 | 2 | 219721165 |  |
| cg16460342 | 1.72E-03 | 12 | 121662577 | *P2RX4* |
| cg02329430 | 1.73E-03 | 15 | 73921385 | *NPTN* |
| cg00695177 | 2.16E-03 | 8 | 8761750 |  |
| cg27203560 | 2.31E-03 | 3 | 193629645 |  |
| cg16400320 | 2.41E-03 | 8 | 144105210 |  |
| cg22764925 | 2.52E-03 | 22 | 24979964 | *GGT1* |
| cg23570810 | 2.69E-03 | 11 | 315102 | *IFITM1* |
| cg22940798 | 2.69E-03 | 6 | 32805554 | *TAP2* |
| cg01044025 | 2.83E-03 | 3 | 151613372 |  |
| cg14043925 | 3.22E-03 | 11 | 3013809 | *NAP1L4* |
| cg27024654 | 3.27E-03 | 5 | 133904674 | *PHF15* |
| cg00959259 | 3.32E-03 | 3 | 122281975 | *PARP9;DTX3L* |
| cg07285983 | 3.41E-03 | 1 | 174844490 | *RABGAP1L* |
| cg23556238 | 3.44E-03 | 12 | 125298876 | *SCARB1* |
| cg13130398 | 3.81E-03 | 1 | 174844397 | *RABGAP1L* |
| cg03816625 | 4.08E-03 | 16 | 12192430 | *SNX29* |
| cg11791770 | 4.25E-03 | 11 | 611791 | *PHRF1* |
| cg25130381 | 4.28E-03 | 1 | 27440721 | *SLC9A1* |
| cg01971407 | 4.44E-03 | 11 | 313624 | *IFITM1* |
| cg13419792 | 4.48E-03 | 19 | 10197996 | *C19orf66* |
| cg15065340 | 4.53E-03 | 3 | 195632915 | *TNK2* |
| cg12110437 | 4.84E-03 | 8 | 144098888 | *LY6E;LOC100133669* |
| cg14392283 | 4.97E-03 | 8 | 144103587 | *LY6E* |
| cg17326313 | 5.21E-03 | 2 | 37383568 | *EIF2AK2* |
| cg21078654 | 5.44E-03 | 15 | 52130305 | *TMOD3* |
| cg03447547 | 5.63E-03 | 14 | 94577039 | *IFI27* |
| cg06473276 | 5.68E-03 | 6 | 39399221 | *KIF6* |
| cg06653140 | 5.99E-03 | 5 | 36157329 | *SKP2* |
| cg05696877 | 6.03E-03 | 1 | 79088769 | *IFI44L* |
| cg12931554 | 6.06E-03 | 8 | 37119428 |  |
| cg02959006 | 6.19E-03 | 16 | 68033589 | *DPEP2* |
| cg12728588 | 6.40E-03 | 1 | 36025489 | *NCDN* |
| cg25115537 | 6.42E-03 | 8 | 123801352 | *ZHX2* |
| cg17932425 | 6.69E-03 | 19 | 17622159 | *PGLS* |
| cg06096336 | 6.70E-03 | 2 | 231989800 | *PSMD1;HTR2B* |
| cg12102235 | 6.77E-03 | 1 | 9047007 |  |
| cg08926253 | 7.08E-03 | 11 | 614761 | *IRF7* |
| cg01254505 | 7.29E-03 | 19 | 17516470 | *BST2* |
| cg12603453 | 7.34E-03 | 6 | 151694679 | *ZBTB2* |
| cg20927242 | 7.98E-03 | 6 | 29692011 | *HLA-F* |
| cg08122652 | 7.98E-03 | 3 | 122281939 | *PARP9;DTX3L* |
| cg00353407 | 8.10E-03 | 1 | 156974025 | *ARHGEF11* |

**Supplementary Table 7. ROC evaluation of DNA methylation levels at DMS found on GO-annotated type I interferon-related genes in patients with various diseases compared with matched controls in CD4+ T cells**

|  |  | **All genes** | ***IFIT1*** | ***IRF7*** | ***MX1*** | ***OAS1*** | ***USP18*** | ***RSAD2*** |
| --- | --- | --- | --- | --- | --- | --- | --- | --- |
| **All four diseases** | **AUC** | 0.90 | 0.82 | 0.78 | 0.80 | 0.80 | 0.78 | 0.81 |
|  | **Sensitivity** | 0.82 | 0.84 | 0.74 | 0.72 | 0.82 | 0.87 | 0.83 |
|  | **Specificity** | 0.82 | 0.71 | 0.75 | 0.78 | 0.66 | 0.62 | 0.69 |
| **GD** | **AUC** | 0.90 | 0.79 | 0.66 | 0.73 | 0.63 | 0.62 | 0.75 |
|  | **Sensitivity** | 0.90 | 0.87 | 0.68 | 0.65 | 0.52 | 0.42 | 0.84 |
|  | **Specificity** | 0.75 | 0.67 | 0.58 | 0.75 | 0.75 | 0.83 | 0.56 |
| **RA** | **AUC** | 1.00 | 0.68 | 0.88 | 0.87 | 0.84 | 0.92 | 0.84 |
|  | **Sensitivity** | 1.00 | 1.00 | 0.83 | 0.83 | 0.83 | 0.75 | 0.92 |
|  | **Specificity** | 1.00 | 0.42 | 0.92 | 0.83 | 0.83 | 1.00 | 0.83 |
| **SLE** | **AUC** | 0.98 | 0.89 | 0.92 | 0.92 | 0.89 | 0.89 | 0.88 |
|  | **Sensitivity** | 1.00 | 1.00 | 1.00 | 1.00 | 0.96 | 1.00 | 1.00 |
|  | **Specificity** | 0.88 | 0.77 | 0.81 | 0.77 | 0.77 | 0.75 | 0.73 |
| **SSc** | **AUC** | 1.00 | 0.75 | 0.73 | 0.86 | 0.82 | 0.78 | 0.77 |
|  | **Sensitivity** | 1.00 | 0.71 | 0.92 | 0.96 | 0.67 | 0.83 | 0.71 |
|  | **Specificity** | 1.00 | 0.75 | 0.54 | 0.75 | 0.83 | 0.67 | 0.71 |

**Supplementary Table 8. ROC evaluation of DNA methylation levels at methylation sites found on *IFI44L* in patients with various diseases compared with matched controls**

|  |  |  | **cg06872964** | **All DMS on *IFI44L*** |
| --- | --- | --- | --- | --- |
| **CD4+ T cells** | **All four diseases** | **AUC** | 0.80 | 0.86 |
|  |  | **Sensitivity** | 0.78 | 0.81 |
|  |  | **Specificity** | 0.74 | 0.80 |
|  | **GD** | **AUC** | 0.58 | 0.77 |
|  |  | **Sensitivity** | 0.35 | 0.61 |
|  |  | **Specificity** | 0.86 | 0.86 |
|  | **RA** | **AUC** | 0.78 | 0.96 |
|  |  | **Sensitivity** | 0.58 | 1.00 |
|  |  | **Specificity** | 0.92 | 0.83 |
|  | **SLE** | **AUC** | 0.89 | 0.95 |
|  |  | **Sensitivity** | 0.98 | 0.98 |
|  |  | **Specificity** | 0.77 | 0.86 |
|  | **SSc** | **AUC** | 0.78 | 0.79 |
|  |  | **Sensitivity** | 0.83 | 0.96 |
|  |  | **Specificity** | 0.75 | 0.58 |
| **CD8+ T cells** | **All two diseases** | **AUC** | 0.65 | 0.75 |
|  |  | **Sensitivity** | 0.93 | 0.89 |
|  |  | **Specificity** | 0.34 | 0.52 |
|  | **GD** | **AUC** | 0.61 | 0.75 |
|  |  | **Sensitivity** | 0.74 | 0.94 |
|  |  | **Specificity** | 0.51 | 0.51 |
|  | **SSc** | **AUC** | 0.73 | 0.73 |
|  |  | **Sensitivity** | 0.50 | 0.92 |
|  |  | **Specificity** | 0.88 | 0.50 |

**Supplementary figure legends**

**Supplementary Fig. 1. Location of methylation sites in relation to gene and CpG island subregions.** Features of all of the methylation sites involved in the differential methylation analysis (red) and the differentially methylated sites identified (blue) are shown in a single figure. (A-B) Proportion of methylation sites related to various gene and CpG island subregions in CD4+ T cells. (C-D) Proportion of methylation sites related to various gene and CpG island subregions in CD8+ T cells.

**Supplementary Fig. 2. Venn diagram of the distribution of differentially methylated sites.** A venn diagram shows the distribution of both the unique and the shared DMS between CD4+ and CD8+ T cells.

**Supplementary Fig. 3. Clustering analysis of CpG sites showing the largest variation (N=50) across CD4+ patient and control groups.** Each column represents a sample, each row represents the methylation level of all the samples involved on one CpG site, and the sample clustering tree appears at the top.

**Supplementary Fig. 4. Forest plot of meta-analysis for methylation level of cg10959651 between GD/RA/SLE/SSc patients and control individuals in CD4+ T cell dataset.** Disease type, standardized mean difference and 95% CI are labelled. The DerSimonian-Laird estimator was selected to conduct combination estimation for the random-effect model.

**Supplementary Fig. 5. ROC curves of the DNA methylation levels at DMS found on all type I interferon-related genes in patients with various diseases compared with matched controls in CD4+ T cells.** (A-D) Curves for patients with GD, RA, SLE and SSc respectively.

**Supplementary Fig. 6. ROC curves of the DNA methylation levels at DMS found on each type I interferon-related gene in GD/RA/SLE/SSc patients compared with matched controls in CD4+ T cells.** (A-F) Curves for *IFIT1*, *IRF7*, *MX1*, *OAS1*, *USP18* and *RSAD2* respectively.

**Supplementary Fig. 7. ROC curves of the DNA methylation levels at DMS found on *IFIT1* in patients with various diseases compared with matched controls in CD4+ T cells.** (A-D) Curves for patients with GD, RA, SLE and SSc respectively.

**Supplementary Fig. 8. ROC curves of the DNA methylation levels at DMS found on *IRF7* in patients with various diseases compared with matched controls in CD4+ T cells.** (A-D) Curves for patients with GD, RA, SLE and SSc respectively.

**Supplementary Fig. 9. ROC curves of the DNA methylation levels at DMS found on *MX1* in patients with various diseases compared with matched controls in CD4+ T cells.** (A-D) Curves for patients with GD, RA, SLE and SSc respectively.

**Supplementary Fig. 10. ROC curves of the DNA methylation levels at DMS found on *OAS1* in patients with various diseases compared with matched controls in CD4+ T cells.** (A-D) Curves for patients with GD, RA, SLE and SSc respectively.

**Supplementary Fig. 11. ROC curves of the DNA methylation levels at DMS found on *USP18* in patients with various diseases compared with matched controls in CD4+ T cells.** (A-D) Curves for patients with GD, RA, SLE and SSc respectively.

**Supplementary Fig. 12. ROC curves of the DNA methylation levels at DMS found on *RSAD2* in patients with various diseases compared with matched controls in CD4+ T cells.** (A-D) Curves for patients with GD, RA, SLE and SSc respectively.

**Supplementary Fig. 13. ROC curves of the DNA methylation level at cg06872964 in all patients compared with matched controls.** (A) Curve for GD/RA/SLE/SSc patients in CD4+ T cells. (B) Curve for GD/SSc patients in CD8+ T cells.

**Supplementary Fig. 14. ROC curves of the DNA methylation level at cg06872964 in patients with various diseases compared with matched controls.** (A-D) Curves for patients with GD, RA, SLE and SSc in CD4+ T cells respectively. (E, F) Curves for patients with GD and SSc in CD8+ T cells respectively.

**Supplementary Fig. 15. ROC curves of the DNA methylation levels at DMS found on *IFI44L* in all patients compared with matched controls.** (A) Curve for GD/RA/SLE/SSc patients in CD4+ T cells. (B) Curve for GD/SSc patients in CD8+ T cells.


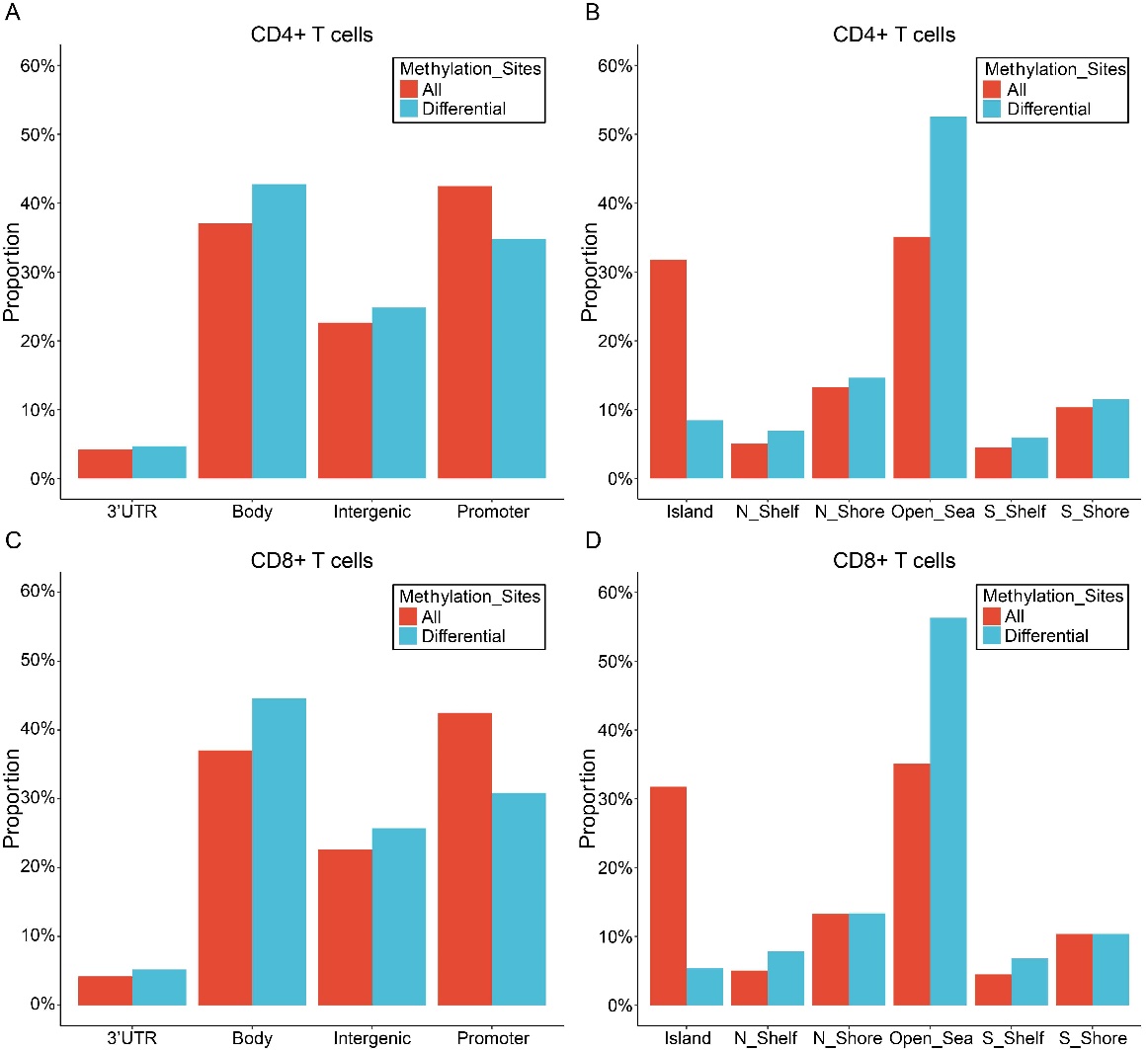


**Supplementary Fig. 1. Location of methylation sites in relation to gene and CpG island subregions.** Features of all of the methylation sites involved in the differential methylation analysis (red) and the differentially methylated sites identified (blue) are shown in a single figure. (A-B) Proportion of methylation sites related to various gene and CpG island subregions in CD4+ T cells. (C-D) Proportion of methylation sites related to various gene and CpG island subregions in CD8+ T cells.


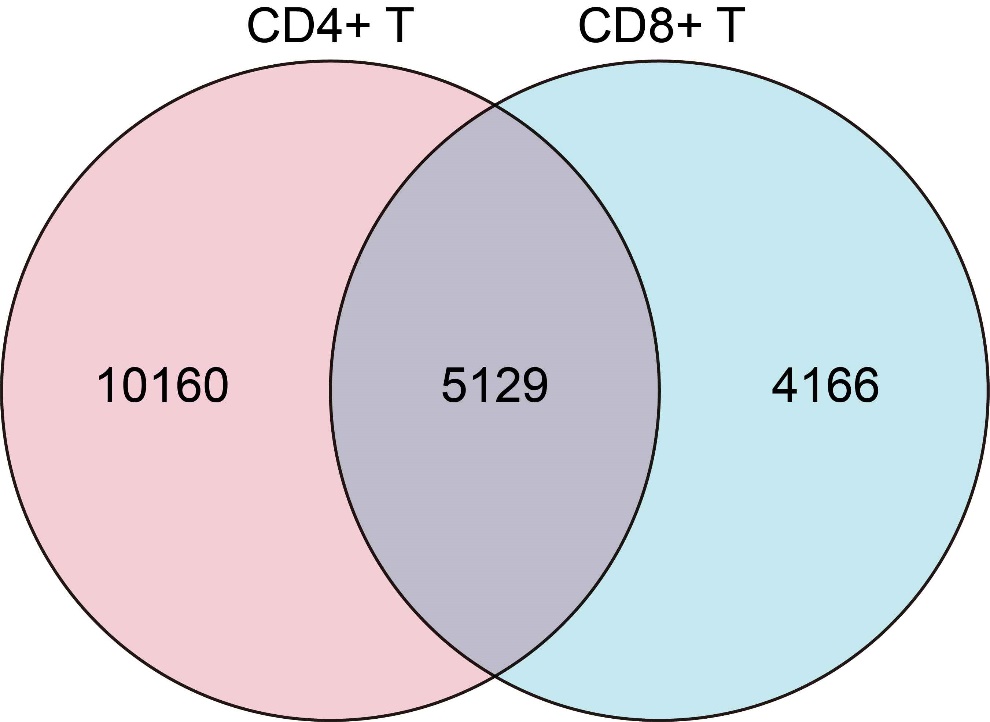


**Supplementary Fig. 2. Venn diagram of the distribution of differentially methylated sites.** A venn diagram shows the distribution of both the unique and the shared DMS between CD4+ and CD8+ T cells.


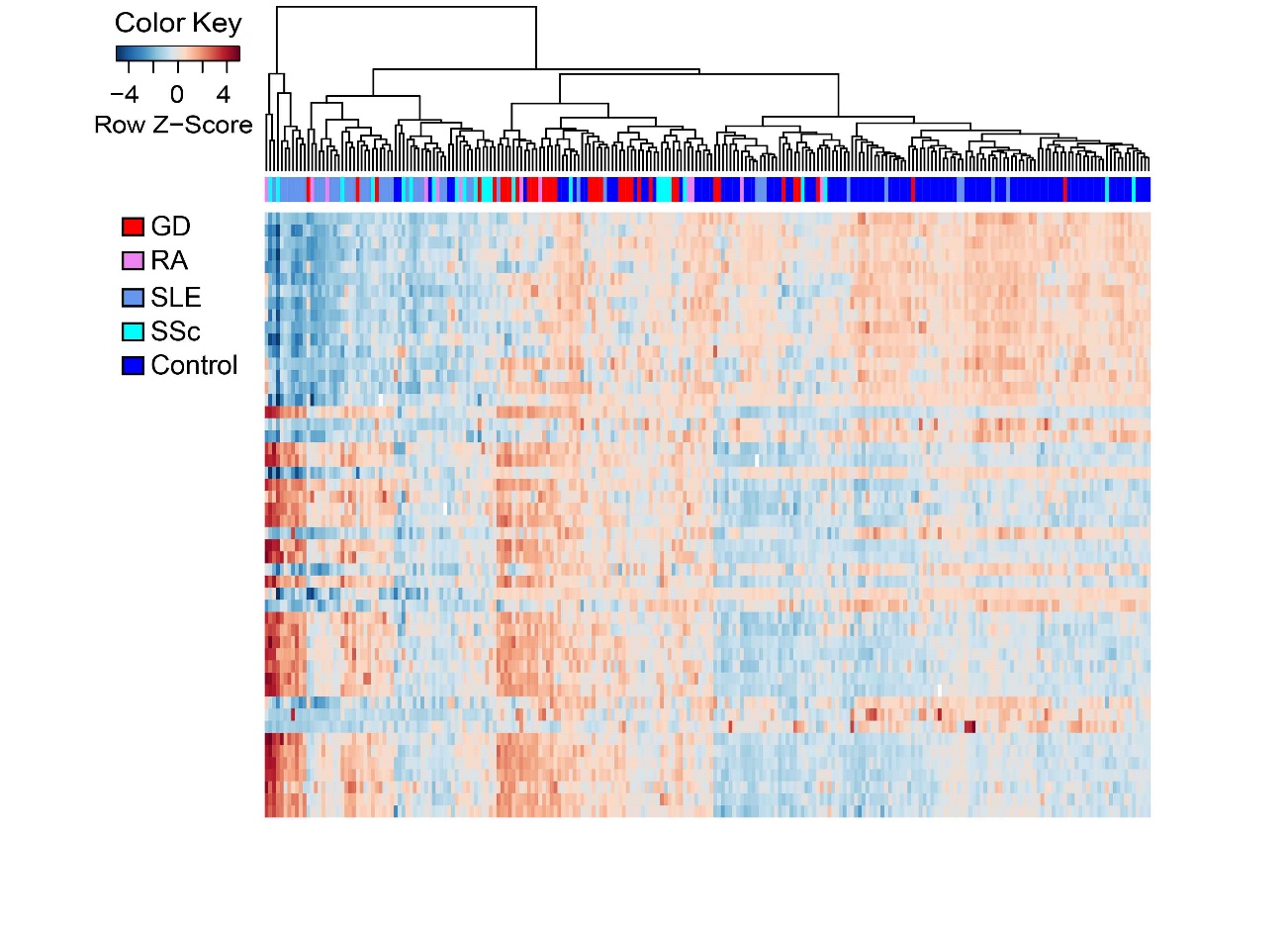


**Supplementary Fig. 3. Clustering analysis of CpG sites showing the largest variation (N=50) across CD4+ patient and control groups.** Each column represents a sample, each row represents the methylation level of all the samples involved on one CpG site, and the sample clustering tree appears at the top.


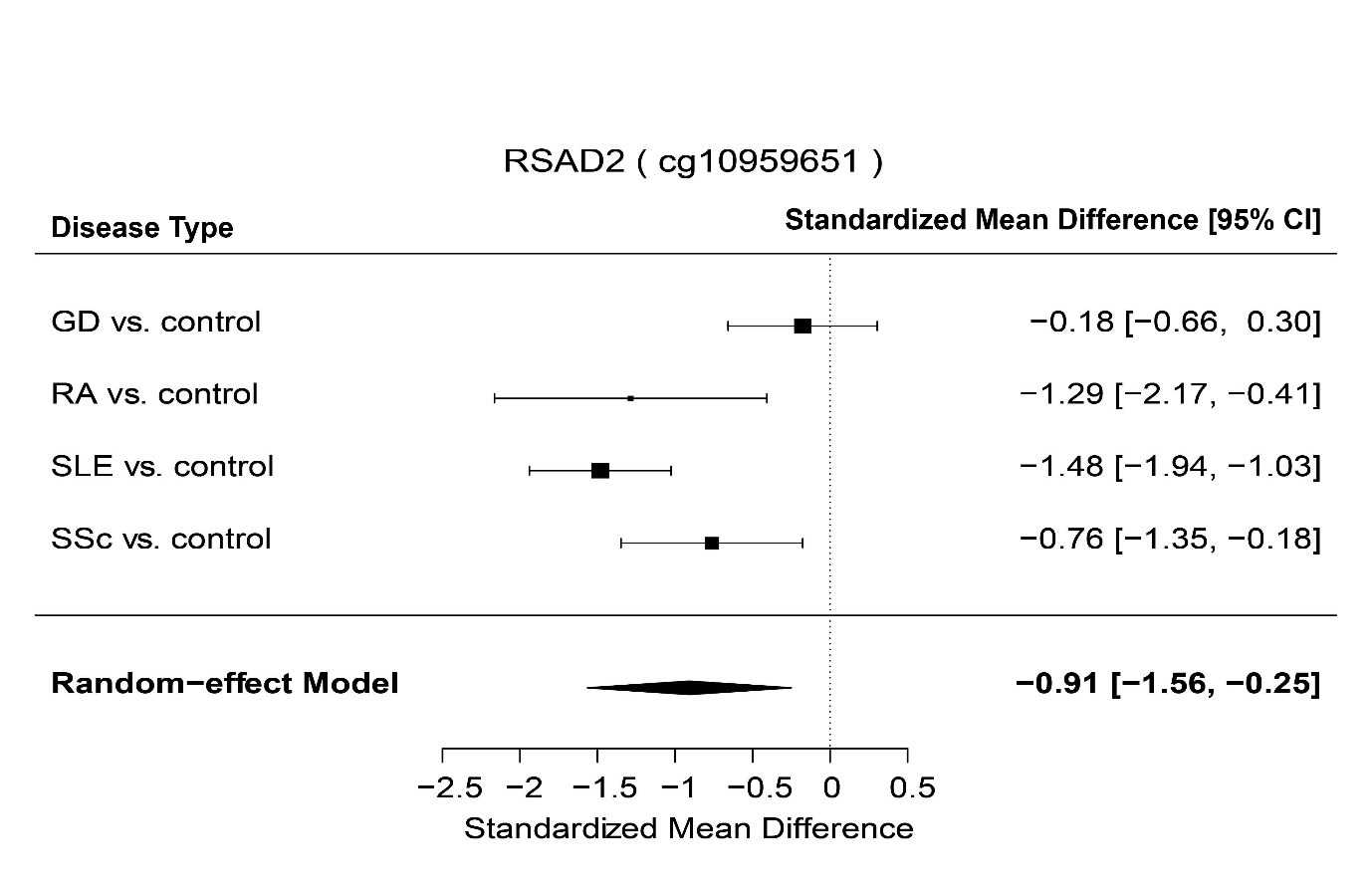


**Supplementary Fig. 4. Forest plot of meta-analysis for methylation level of cg10959651 between GD/RA/SLE/SSc patients and control individuals in CD4+ T cell dataset.** Disease type, standardized mean difference and 95% CI are labelled. The DerSimonian-Laird estimator was selected to conduct combination estimation for the random-effect model.


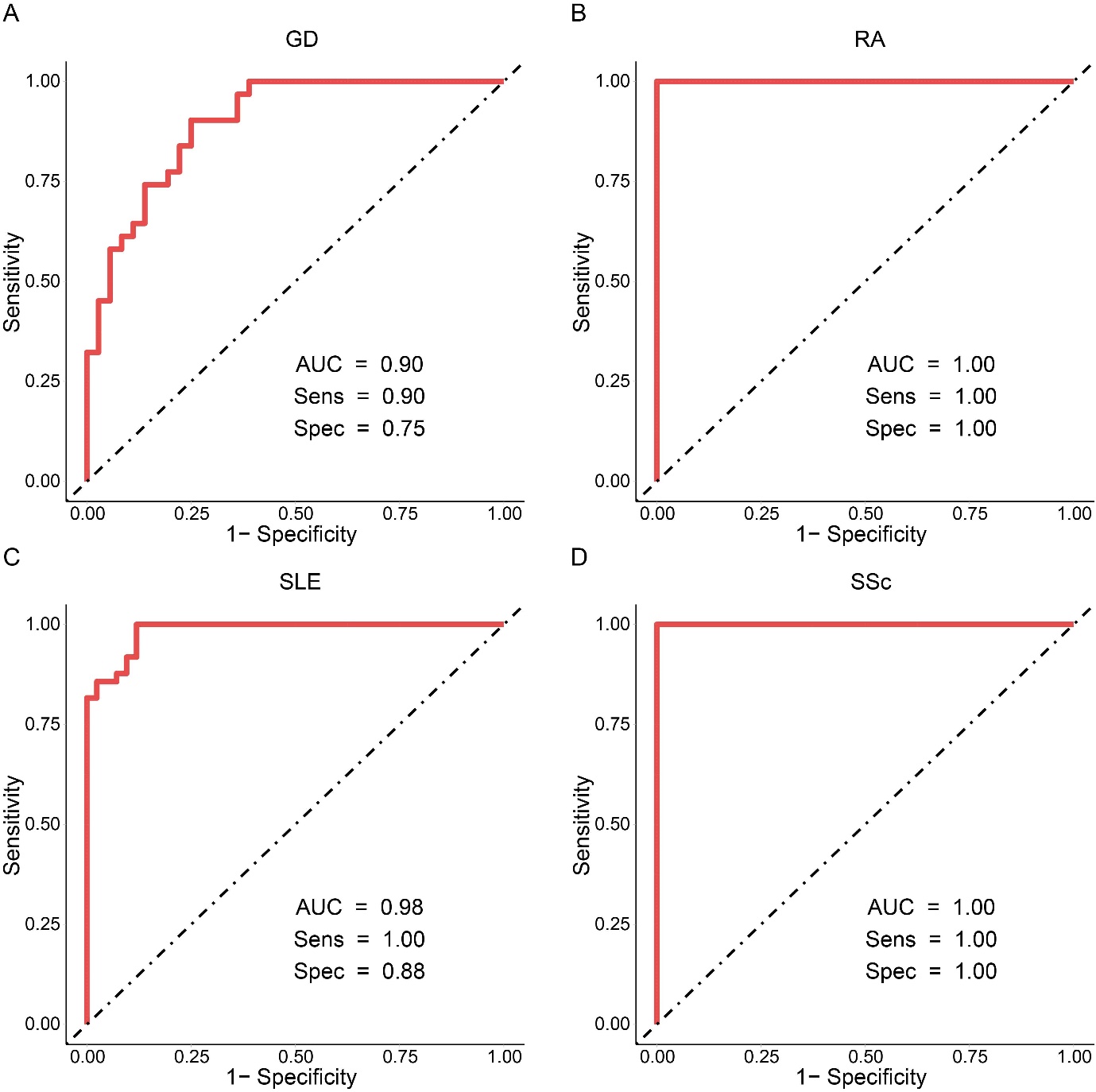


**Supplementary Fig. 5. ROC curves of the DNA methylation levels at DMS found on all type I interferon-related genes in patients with various diseases compared with matched controls in CD4+ T cells.** (A-D) Curves for patients with GD, RA, SLE and SSc respectively.


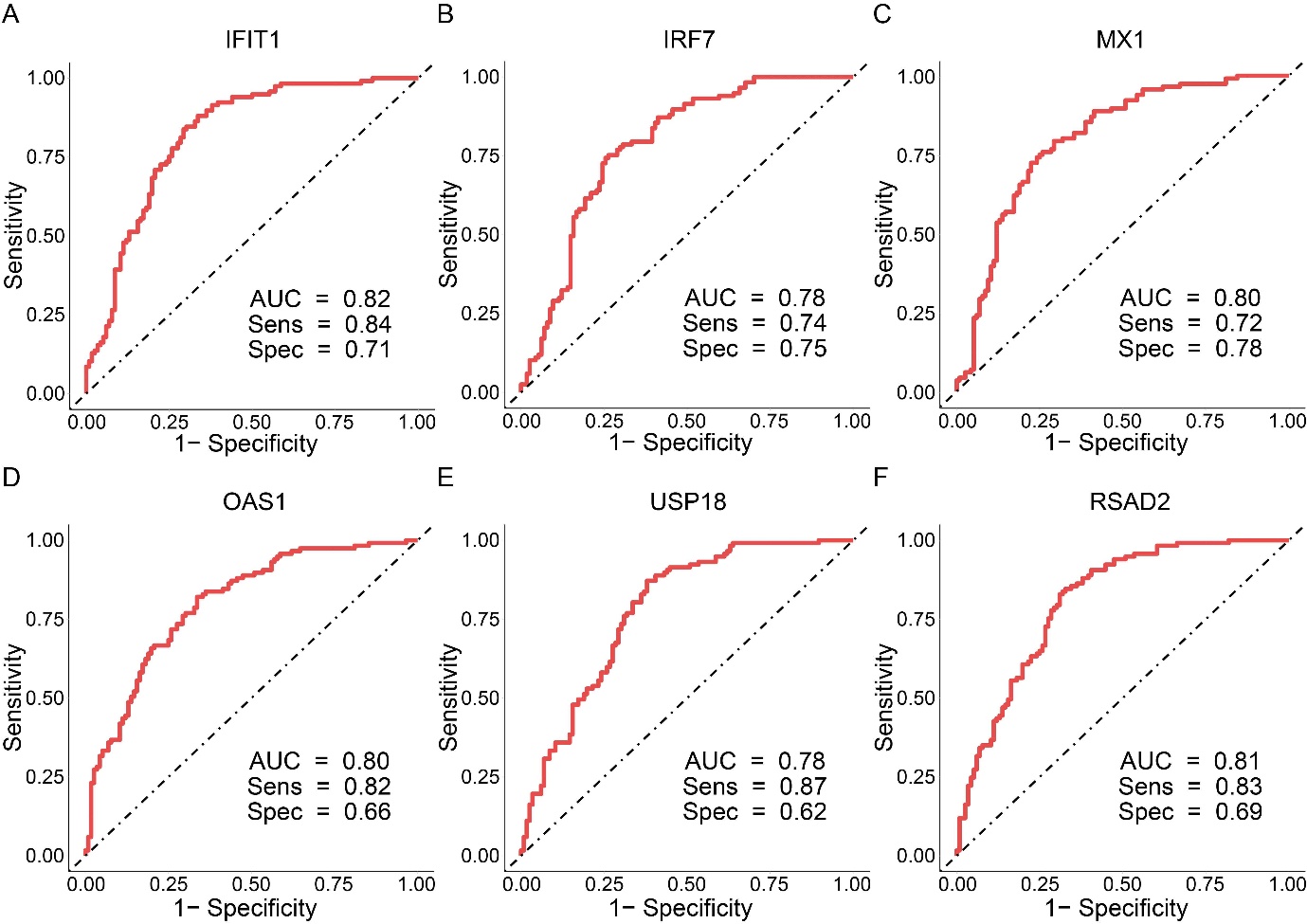


**Supplementary Fig. 6. ROC curves of the DNA methylation levels at DMS found on each type I interferon-related gene in GD/RA/SLE/SSc patients compared with matched controls in CD4+ T cells.** (A-F) Curves for *IFIT1*, *IRF7*, *MX1*, *OAS1*, *USP18* and *RSAD2* respectively.


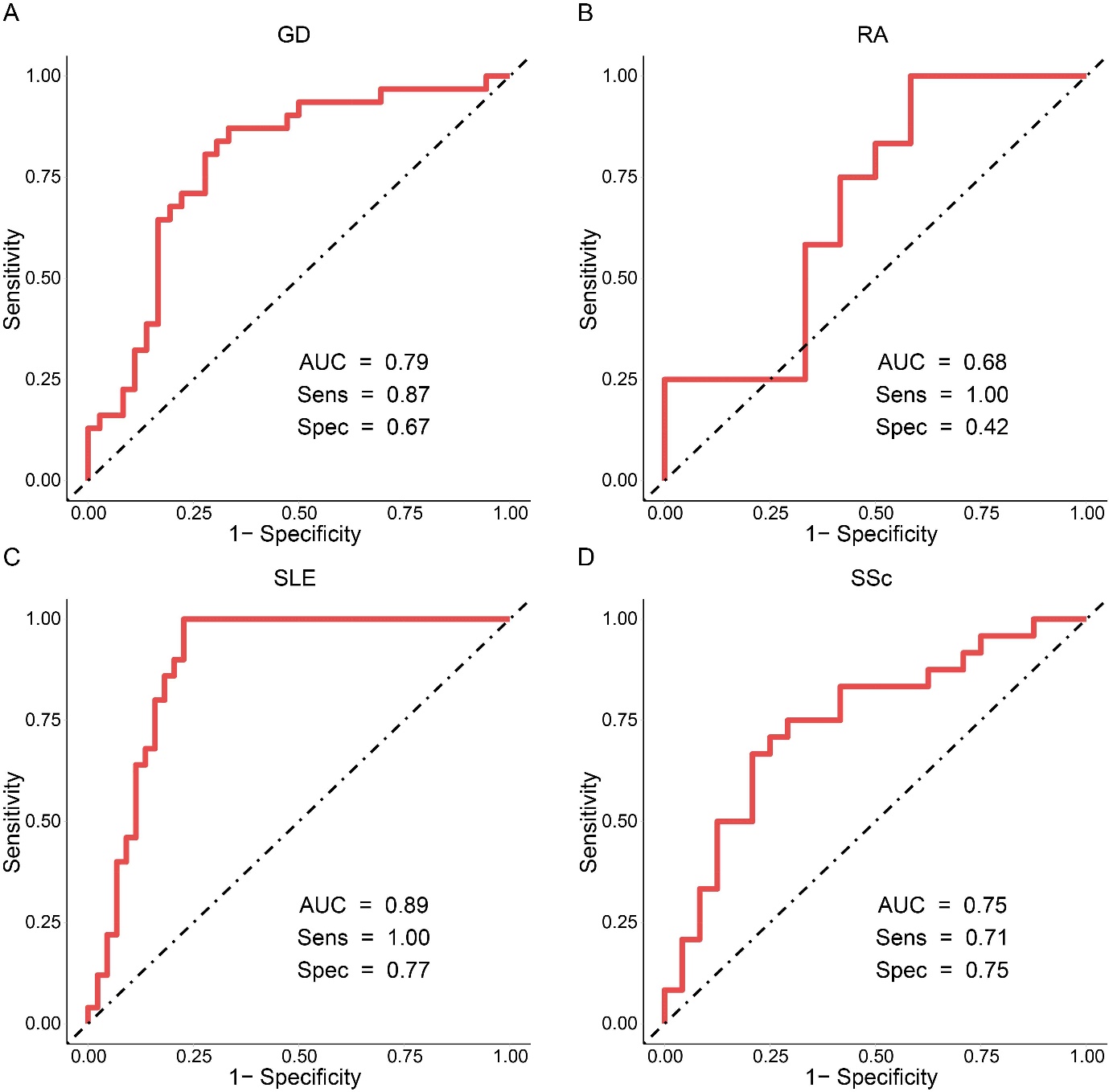


**Supplementary Fig. 7. ROC curves of the DNA methylation levels at DMS found on *IFIT1* in patients with various diseases compared with matched controls in CD4+ T cells.** (A-D) Curves for patients with GD, RA, SLE and SSc respectively.


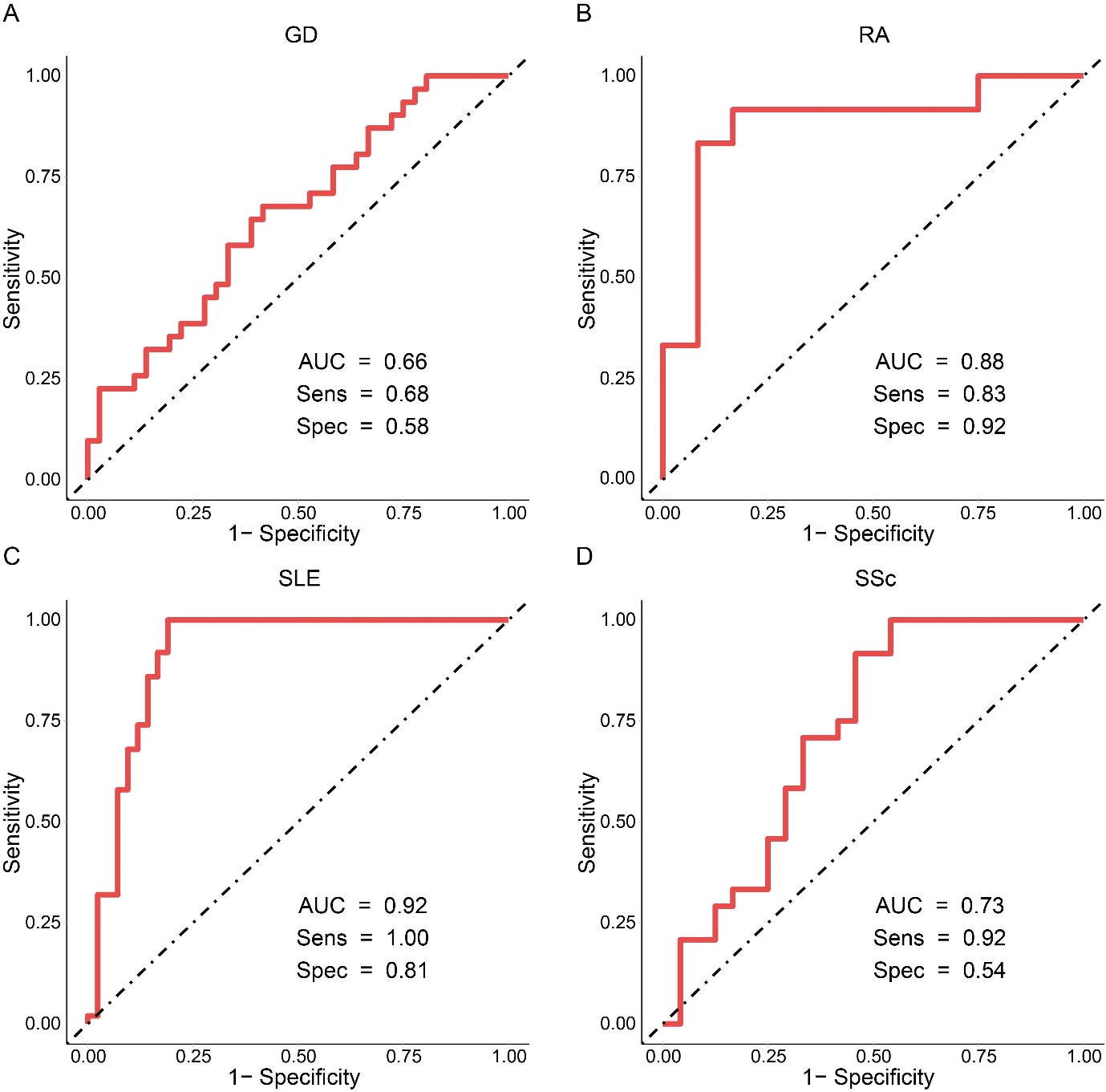


**Supplementary Fig. 8. ROC curves of the DNA methylation levels at DMS found on *IRF7* in patients with various diseases compared with matched controls in CD4+ T cells.** (A-D) Curves for patients with GD, RA, SLE and SSc respectively.


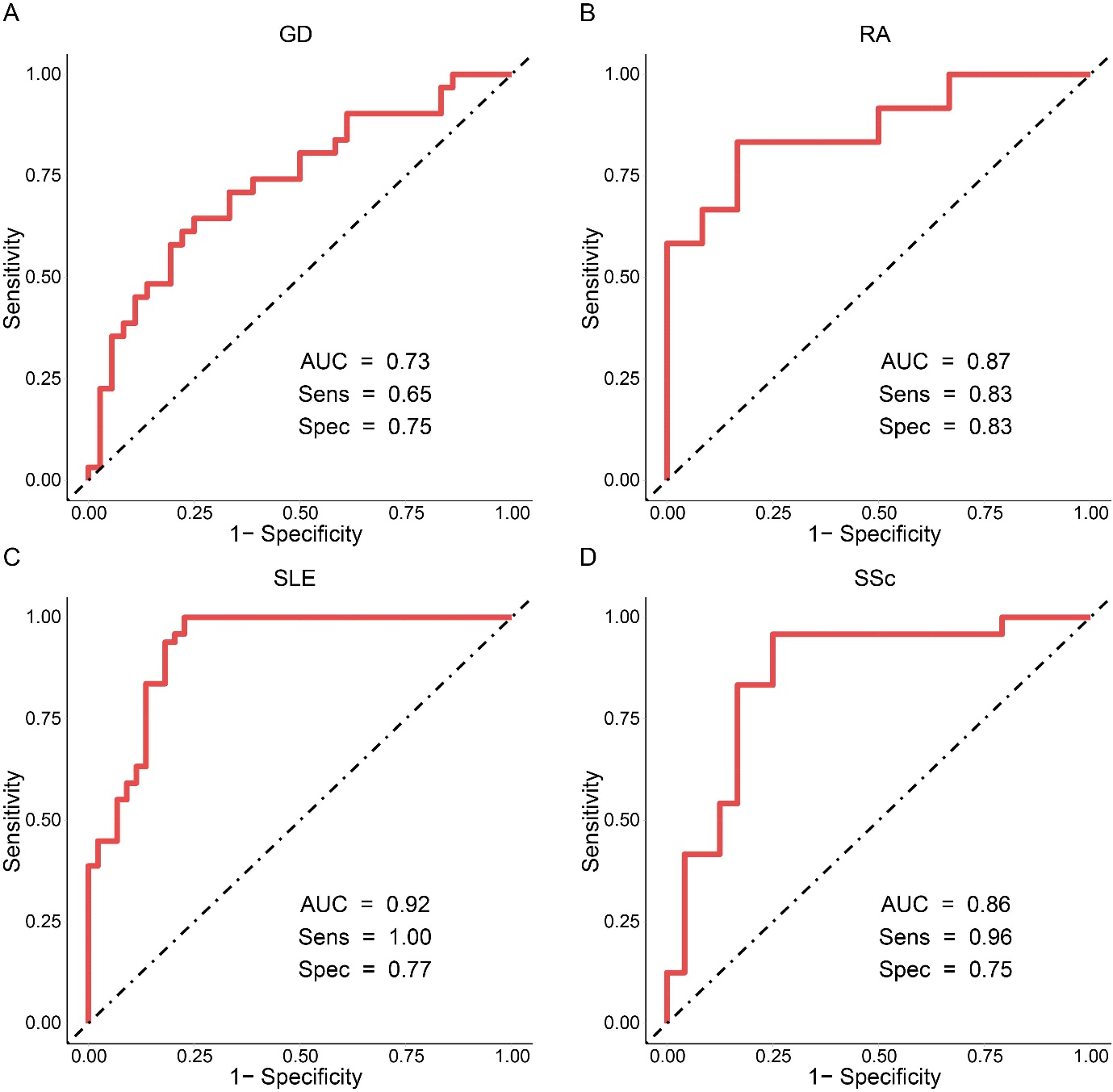


**Supplementary Fig. 9. ROC curves of the DNA methylation levels at DMS found on *MX1* in patients with various diseases compared with matched controls in CD4+ T cells.** (A-D) Curves for patients with GD, RA, SLE and SSc respectively.


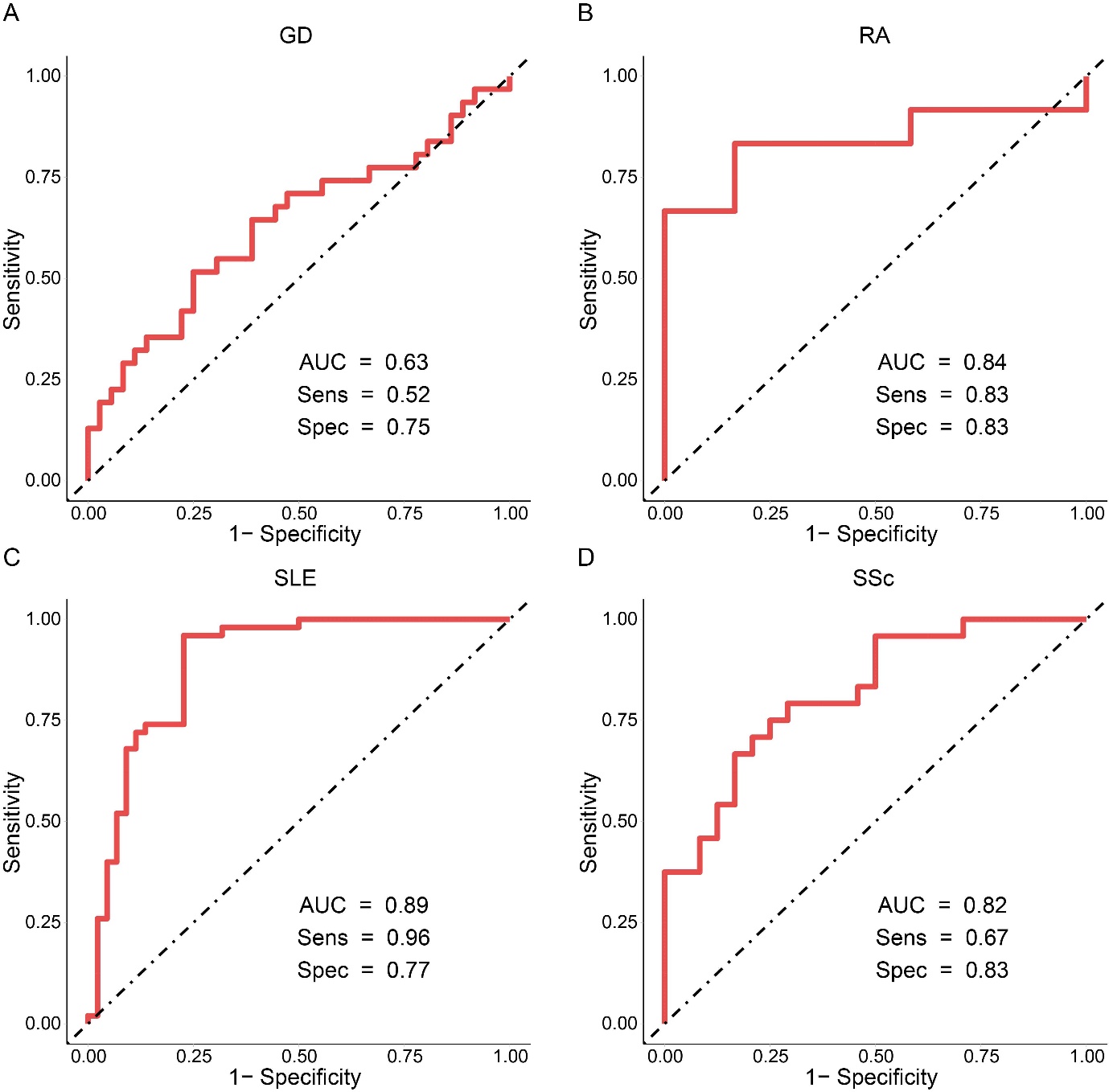


**Supplementary Fig. 10. ROC curves of the DNA methylation levels at DMS found on *OAS1* in patients with various diseases compared with matched controls in CD4+ T cells.** (A-D) Curves for patients with GD, RA, SLE and SSc respectively.


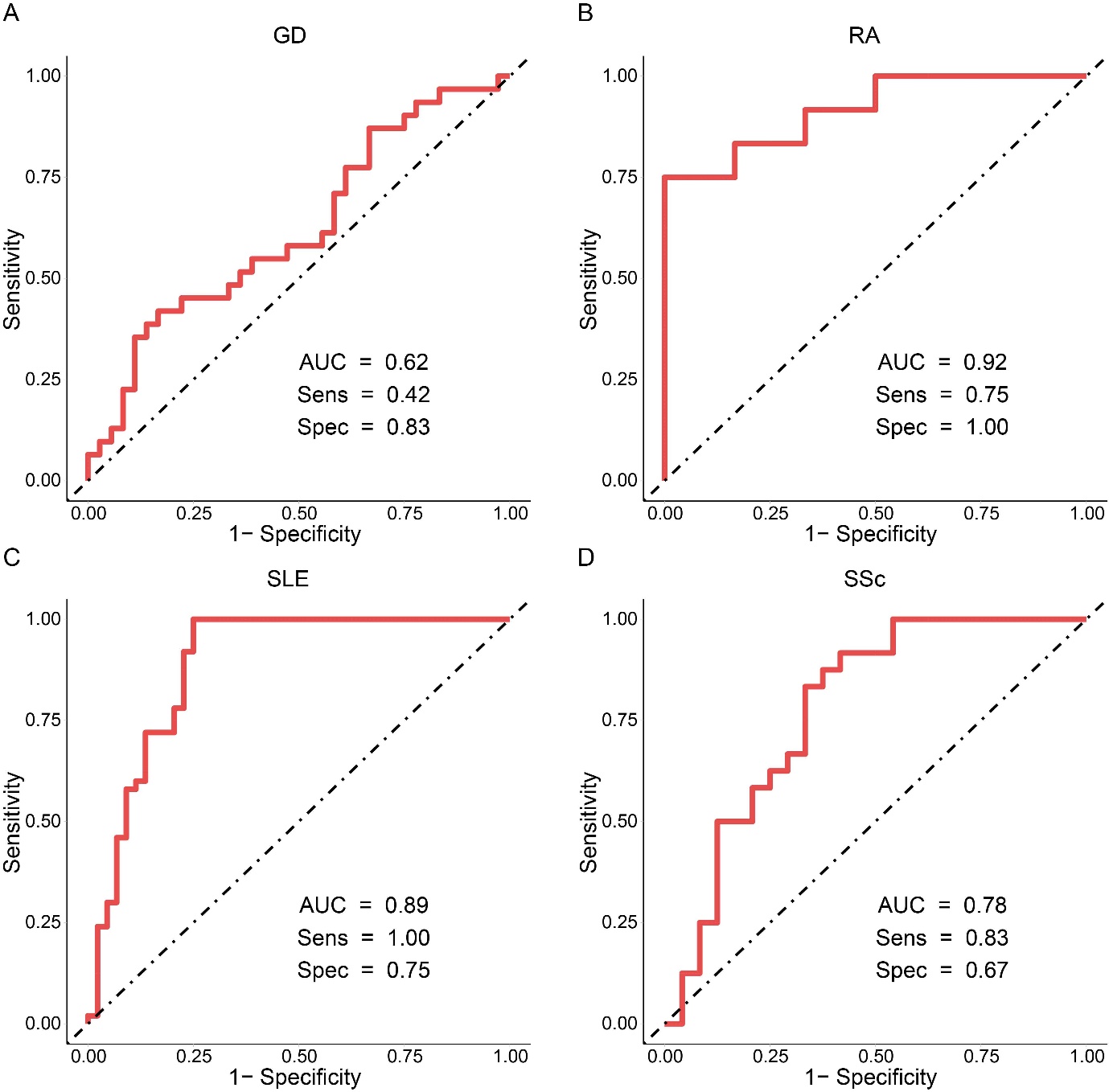


**Supplementary Fig. 11. ROC curves of the DNA methylation levels at DMS found on *USP18* in patients with various diseases compared with matched controls in CD4+ T cells.** (A-D) Curves for patients with GD, RA, SLE and SSc respectively.


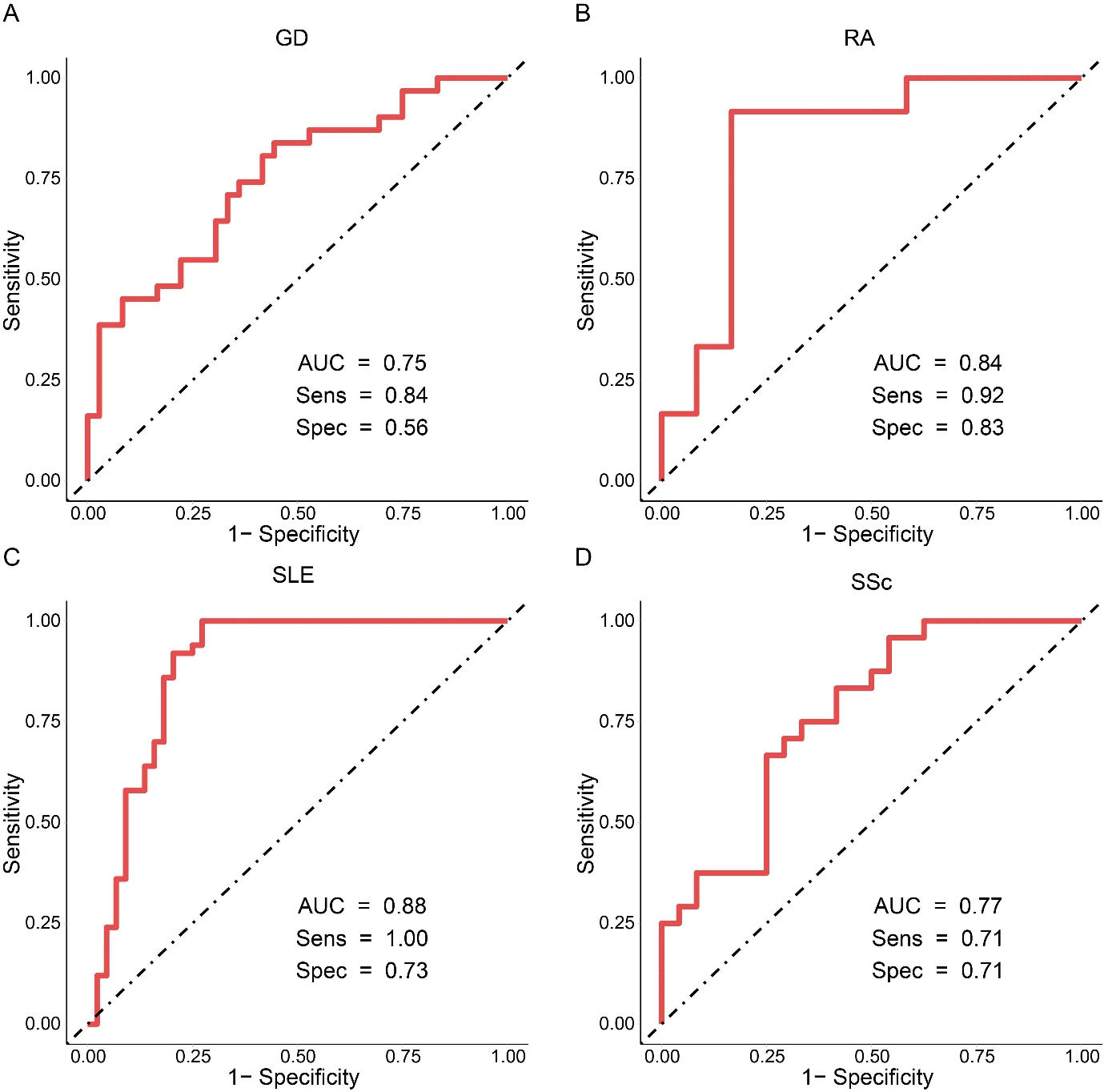


**Supplementary Fig. 12. ROC curves of the DNA methylation levels at DMS found on *RSAD2* in patients with various diseases compared with matched controls in CD4+ T cells.** (A-D) Curves for patients with GD, RA, SLE and SSc respectively.


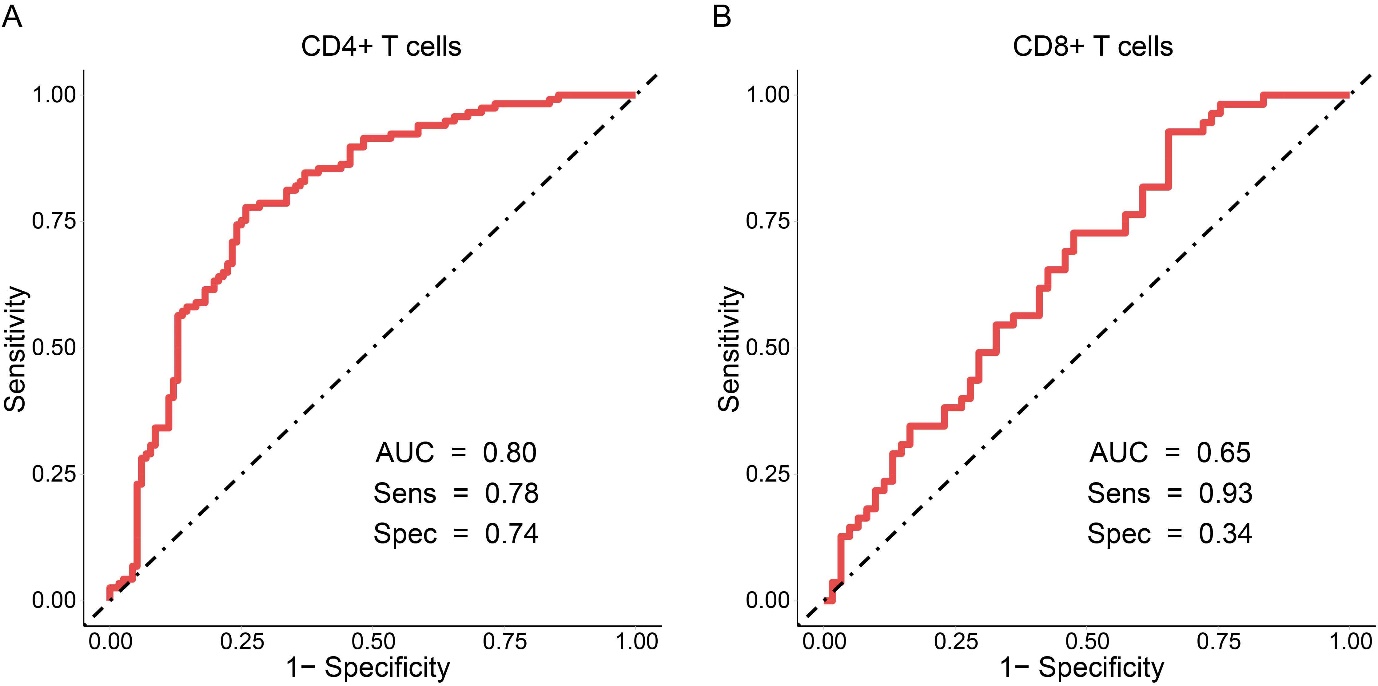


**Supplementary Fig. 13. ROC curves of the DNA methylation level at cg06872964 in all patients compared with matched controls.** (A) Curve for GD/RA/SLE/SSc patients in CD4+ T cells. (B) Curve for GD/SSc patients in CD8+ T cells.


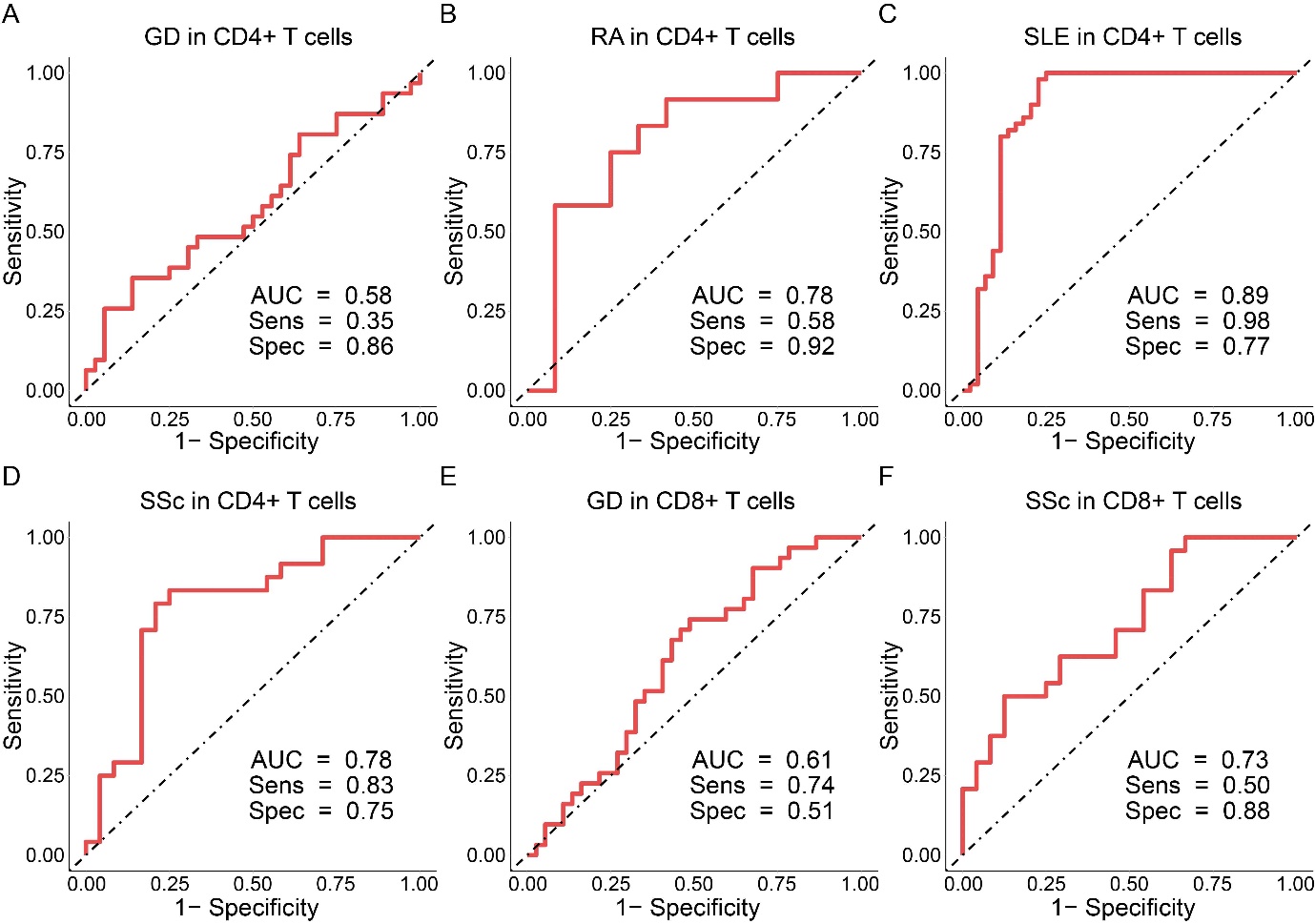


**Supplementary Fig. 14. ROC curves of the DNA methylation level at cg06872964 in patients with various diseases compared with matched controls.** (A-D) Curves for patients with GD, RA, SLE and SSc in CD4+ T cells respectively. (E, F) Curves for patients with GD and SSc in CD8+ T cells respectively.


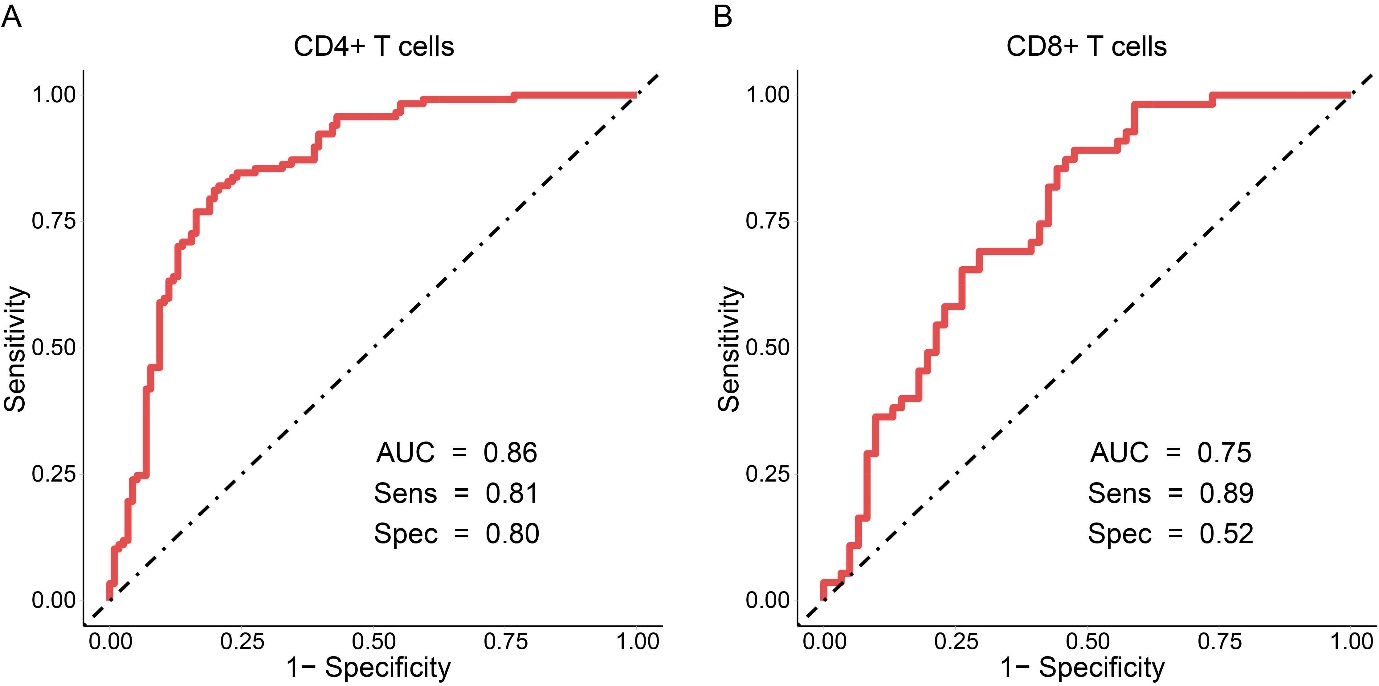


**Supplementary Fig. 15. ROC curves of the DNA methylation levels at DMS found on *IFI44L* in all patients compared with matched controls.** (A) Curve for GD/RA/SLE/SSc patients in CD4+ T cells. (B) Curve for GD/SSc patients in CD8+ T cells.
